# A Mobile Health Salt Reduction Intervention for People With Hypertension: Results of a Feasibility Randomized Controlled Trial

**DOI:** 10.2196/26233

**Published:** 2021-10-21

**Authors:** Sarah Payne Riches, Carmen Piernas, Paul Aveyard, James P Sheppard, Mike Rayner, Charlotte Albury, Susan A Jebb

**Affiliations:** 1 Nuffield Department of Primary Care Health Sciences University of Oxford Oxford United Kingdom; 2 National Institute for Health Research Oxford Biomedical Research Centre Oxford University Hospitals Oxford United Kingdom; 3 Nuffield Department of Population Health University of Oxford Oxford United Kingdom

**Keywords:** salt reduction, behavior change, mobile health, mHealth, smartphone app, mobile phone

## Abstract

**Background:**

A high-salt diet is a risk factor for hypertension and cardiovascular disease; therefore, reducing dietary salt intake is a key part of prevention strategies. There are few effective salt reduction interventions suitable for delivery in the primary care setting, where the majority of the management and diagnosis of hypertension occurs.

**Objective:**

The aim of this study is to assess the feasibility of a complex behavioral intervention to lower salt intake in people with elevated blood pressure and test the trial procedures for a randomized controlled trial to investigate the intervention’s effectiveness.

**Methods:**

This feasibility study was an unblinded, randomized controlled trial of a mobile health intervention for salt reduction versus an advice leaflet (control). The intervention was developed using the Behavior Change Wheel and comprised individualized, brief advice from a health care professional with the use of the SaltSwap app. Participants with an elevated blood pressure recorded in the clinic were recruited through primary care practices in the United Kingdom. Primary outcomes assessed the feasibility of progression to a larger trial, including follow-up attendance, fidelity of intervention delivery, and app use. Secondary outcomes were objectively assessed using changes in salt intake (measured via 24-hour urine collection), salt content of purchased foods, and blood pressure. Qualitative outcomes were assessed using the think-aloud method, and the process outcomes were evaluated.

**Results:**

A total of 47 participants were randomized. All progression criteria were met: follow-up attendance (45/47, 96%), intervention fidelity (25/31, 81%), and app use (27/31, 87%). There was no evidence that the intervention significantly reduced the salt content of purchased foods, salt intake, or blood pressure; however, this feasibility study was not powered to detect changes in secondary outcomes. Process and qualitative outcomes demonstrated that the trial design was feasible and the intervention was acceptable to both individuals and practitioners and positively influenced salt intake behaviors.

**Conclusions:**

The intervention was acceptable and feasible to deliver within primary care; the trial procedures were practicable, and there was sufficient signal of potential efficacy to change salt intake. With some improvements to the intervention app, a larger trial to assess intervention effectiveness for reducing salt intake and blood pressure is warranted.

**Trial Registration:**

International Standard Randomized Controlled Trial Number (ISRCTN): 20910962; https://www.isrctn.com/ISRCTN20910962

## Introduction

### Background

Cardiovascular disease (CVD) is the leading cause of premature mortality worldwide [1], and one of the leading risk factors for CVD is hypertension [2-4]. Hypertension affects a quarter of the adult population in the United Kingdom [5]. A high-salt diet increases blood pressure [6-14], and reducing salt intake has consistently been shown to reduce blood pressure in those with hypertension [15]. Recent estimates have identified a high-sodium diet as the largest *dietary* risk factor for global mortality [16]. Therefore, reducing dietary salt intake is a key factor in CVD prevention. In the United Kingdom, for example, adults consume, on average, 40% more salt than that recommended by the national and international dietary guidelines [17,18], with the majority of salt derived from processed foods rather than salt added during cooking or at the table [19].

A population-level salt reduction strategy was implemented in the United Kingdom in 2003, which achieved some success in reducing the population-level salt intake [20]. However, there has been no significant reduction in average population salt intake since 2009 [17]. Despite good evidence for the effectiveness of individual-level interventions centered on dietary advice and acquisition of skills to achieve and maintain a low-salt diet [7,8,21], effective interventions tend to be intensive or require specialist staff. There are few interventions suitable for delivery in the primary care setting [22], where the majority of the management and diagnosis of hypertension occurs [23]. In the United Kingdom, clinicians are encouraged, through national guidelines, to advise reduced salt intake when managing hypertension, but they have no tools, structured advice, or guidance for doing so effectively [23,24]. *Brief interventions* are those which can be delivered by clinical staff without specialist nutrition or behavior change knowledge and require minimal health professional contact [25]. Such interventions could offer significant and sustainable impacts with less participant burden and greater reach than more intensive approaches. Behaviorally informed, brief interventions for dietary behavior change have been used successfully for weight loss or maintenance and cholesterol reduction, using techniques such as self-regulation or habit control to drive behavior change [26-30] but not, to our knowledge, for salt reduction in a clinically at-risk population.

Mobile apps have been shown to support dietary changes for weight loss or increased fruit and vegetable consumption [31,32]. Although high-quality evidence for the use of mobile apps in salt reduction is limited, recent studies have shown promising effects. A study investigating a daily diet monitoring app in a university population in the United States showed a significant reduction in salt intake over 4 weeks [33]; a study using an app to encourage the purchase of lower-salt food in New Zealand found a significant reduction in recorded salt purchases but no evidence of reductions in the overall salt intake [34]. A further study in the United States, using geofencing to send targeted messages on salt reduction based on location, demonstrated a reduction in the estimated salt intake from spot urine samples but no significant change in daily urinary sodium excretion [35]. The potential benefits of using apps to deliver health interventions include the low relative cost of delivery, the opportunity to deliver at scale, the low cost compared with face-to-face interventions, the flexibility to allow tailoring to individuals, and the ability to deliver an intervention with consistency. The fact that individuals often have their devices with them when they are undertaking the target behavior could make the intervention more salient. We propose that mobile apps could complement and augment brief interventions for salt reduction delivered in primary care—the principal setting for the diagnosis and management of hypertension in the United Kingdom.

### Objectives

We developed a behaviorally informed brief intervention, incorporating a mobile app, to motivate and support individuals to reduce their salt intake by choosing lower-salt foods when grocery shopping. This paper describes the design of the SaltSwap intervention and reports the results of a randomized controlled trial (RCT) that incorporates qualitative and process outcomes. The trial examined whether the intervention is feasible to deliver in primary care and if it has the potential to reduce salt intake and blood pressure and tested the procedures for a future effectiveness trial.

## Methods

### Study Design and Participants

This feasibility study was an individually randomized, parallel, 2-arm, controlled trial. After providing consent and completing the baseline assessment, participants entered a 2-week run-in period, and those who provided a baseline urine sample and grocery shopping data during this period were randomized. After the 6-week intervention period, the participants attended a single follow-up visit. The study was reviewed and approved on March 17, 2017, by the National Health Service Research Ethics Committee and the Health Research Authority (reference 17/SC/0098). The trial protocol was registered on April 5, 2017. The study methods are published elsewhere [36] and are summarized below.

### Recruitment

Participants were recruited through 5 general practitioner (GP) surgeries in Oxfordshire, United Kingdom. GP surgery electronic health records were searched for adults with a recent blood pressure reading (systolic blood pressure in the past 2 years above 130 mm Hg if currently prescribed a stable-dose, antihypertensive medication or above 140 mm Hg if not prescribed medication), and these patients were invited by letter to participate. Letters included a web link to web-based study information. Major exclusion criteria included the following: secondary, previous accelerated or malignant hypertension, currently being assessed for diagnosis of hypertension; existing or recent cardiovascular conditions; or following a clinician-supervised diet or a restricted diet. The principal investigator screened interested participants by phone according to the full inclusion and exclusion [36] criteria and provided study information. Eligible participants were booked into a baseline study visit, where a study researcher provided written study information and confirmed consent in person.

### Randomization

Participants were randomly allocated in a 2:1 ratio (intervention:control) using a computer-generated allocation sequence stratified by GP surgery. An independent researcher generated a random number sequence using a web-based random sequence generator and informed the principal investigator of the intervention allocation for each participant. Participants and investigators were unaware of the treatment allocation unaware of the treatment allocation prior to participant consent. Owing to the nature of the intervention and trial procedures, it was not possible to blind participants, clinicians delivering the intervention, or the outcome assessor to the treatment group.

### Intervention

**Figure 1:**
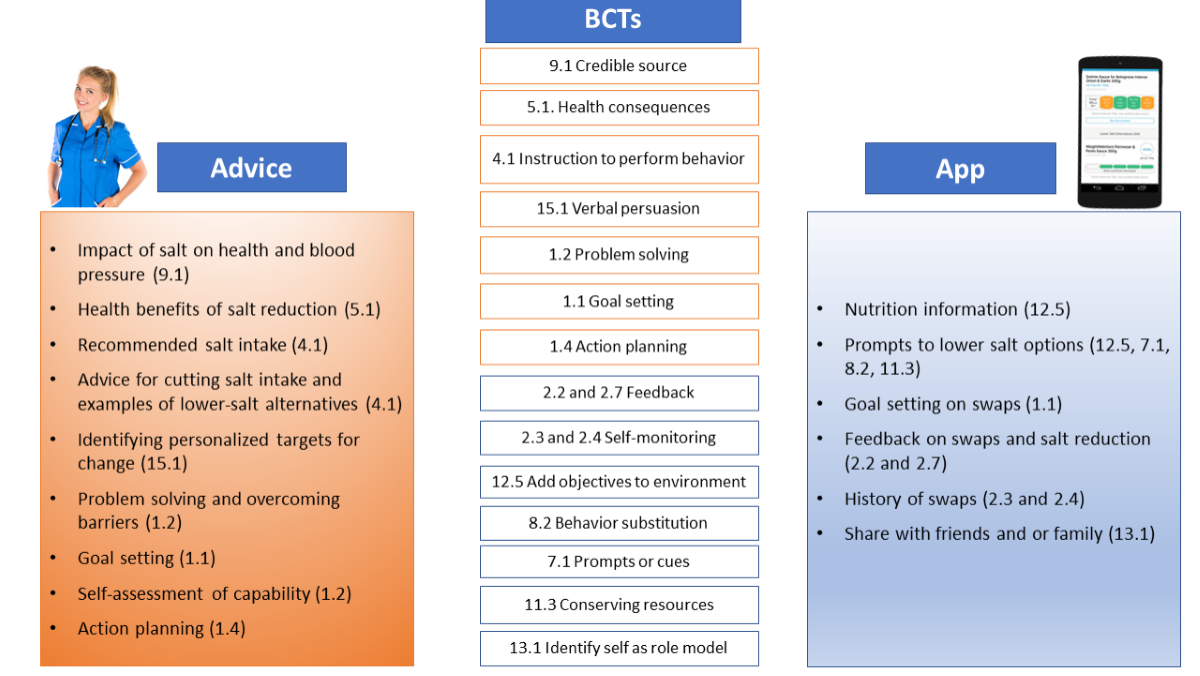
The SaltSwap intervention and included behavior change techniques. BCT: behavior change technique.

**Figure 2:**
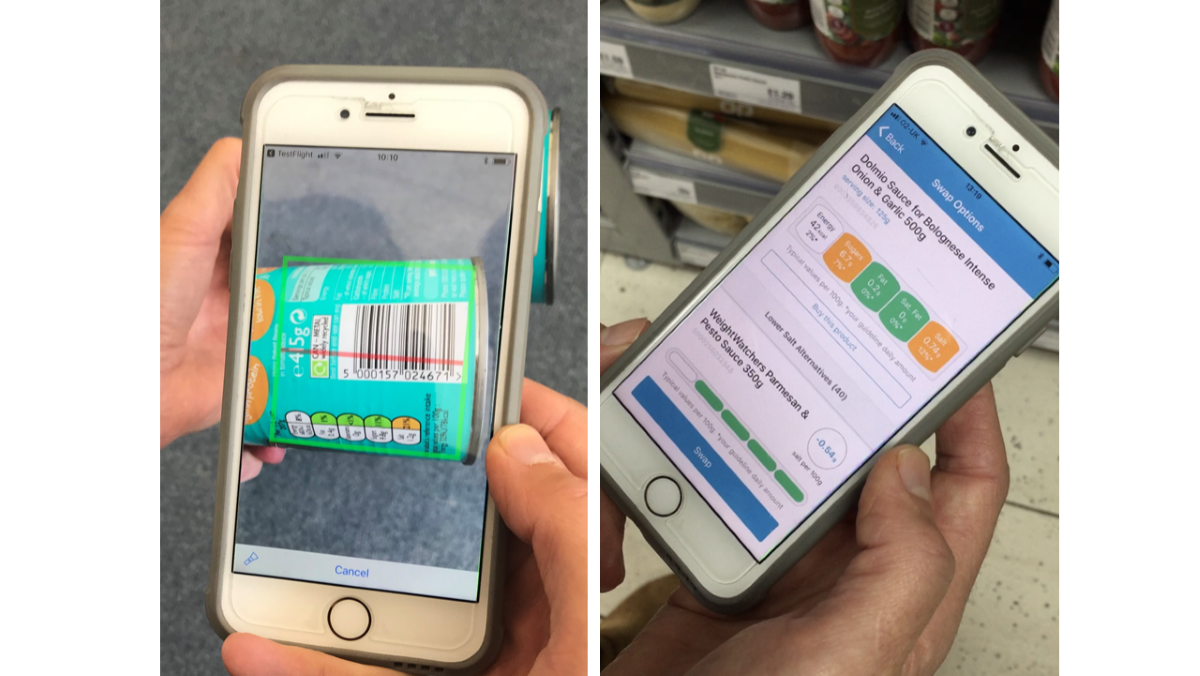
The SaltSwap app screenshots.

The SaltSwap intervention aimed to reduce dietary salt intake by encouraging individuals to swap to lower-salt alternatives when grocery shopping, buy fewer high-salt foods, and use less salt when cooking or at the table. We designed the intervention using the Behavior Change Wheel, a theoretical framework widely used to develop health behavior change interventions [37] (Multimedia Appendix 1 [38]). We used a co-design approach, recruiting a panel of individuals diagnosed with hypertension via a preexisting database of volunteers and advertisements in GP surgeries in Oxford. This panel informed the behavioral analysis, intervention content, mode of delivery, and key questions for intervention evaluation. Panel members also reviewed the final intervention design and conducted the initial pilot testing. The intervention comprised behavioral advice and support, provided by a health care professional (typically a nurse or health care assistant), in a 20-minute face-to-face intervention visit. This visit aimed to educate and persuade individuals about the importance of salt reduction and how they can reduce their salt intake and provide the motivation to do this using established behavior change techniques (BCTs; Figure 1) [38]. These BCTs were largely drawn from the BCT taxonomy groups *goals and planning* and *feedback and monitoring*, which have significant evidence to support their effectiveness in dietary behavior change [38-40]. The intervention visit also introduced the SaltSwap app to help individuals identify lower-salt options when grocery shopping, provide feedback on swaps made, and enable users to share successful swaps with their social network (Figure 2; Multimedia Appendix 2 [41,42]). The app enabled users to record their purchased products and any swaps made. Health care professionals were trained in a 1-hour face-to-face session covering the intervention content, theoretical background, and familiarity with the app and were provided training videos for future reference. All other clinical care was continued as usual.

### Comparator

Participants randomized to the control arm received *usual care* in the form of a postal copy of the publicly available British Heart Foundation Cut Down on Salt booklet [43] or its successor Taking Control of Salt [44]. On completion of their follow-up visit, control participants were given the SaltSwap booklet and were shown how to download the SaltSwap app. Control participants were not aware that they would receive the intervention materials until the end of the follow-up visit. All other clinical care was continued as usual in both arms.

### Procedures

Participants attended a baseline study visit where demographic information, medical history (including medications), and clinical measures (height, weight, and blood pressure) were collected. Participants completed a questionnaire on their shopping behaviors and were asked to collect a single 24-hour urine sample for urinary sodium assessment (written instructions provided). They were also asked to collect household grocery shopping receipts and record all grocery purchases using a purchase scanning app for a 2-week baseline run-in period (Multimedia Appendix 3). Only participants who returned baseline data were randomized. Participants were informed of their baseline blood pressure readings but no other baseline measures.

Participants randomized to the intervention group were booked an intervention appointment with a health care professional; control participants were sent a copy of the control booklet by post. The intervention appointment was audio recorded to assess the intervention fidelity. All participants were asked to record their grocery shopping purchases and collect grocery shopping receipts over a 6-week intervention period. SMS text messaging prompts were sent to remind participants to collect their shopping data if the app was not accessed within 7 days and when the app was not used for 10 days after the first use. Participants were scheduled for a follow-up visit with a researcher 6 weeks post randomization, where blood pressure was measured. Participants were asked to collect a second 24-hour urine sample, on any week or weekend day, within 7 days of this appointment. At this follow-up visit, participants completed a questionnaire about changes in their dietary salt knowledge and behaviors.

A convenience sample of intervention participants was invited to participate in an accompanied shopping trip whereby the lead author observed them doing their usual supermarket shopping, using the think-aloud method that attempts to access and record participants’ inner speech, the *transformation of thought processes into words* [45]. The think-aloud method has been widely used in the design and evaluation of digital interventions [46,47] and to explore consumers’ food choice behaviors [48-51]. The accompanied shopping trip was followed by a one-on-one interview about the participants’ experiences of the intervention. Data were captured through audio recordings, which were transcribed by an external transcriber and supplemented by field notes.

### Outcome Measures

#### Primary Outcomes

The primary outcomes were prespecified progression criteria:

1. Follow-up rate: At least 65% of the randomized participants attend the single follow-up visit.
2. Fidelity of intervention delivery: Health care professionals deliver at least 4 of the 6 prespecified essential intervention elements during the advice session.
3. Use of the SaltSwap app: At least 50% of the randomized participants use the SaltSwap app to scan products on at least 1 occasion in the first month.

The principal investigator and a second researcher coassessed fidelity of delivery of the first 5 intervention sessions using audio recordings of the intervention delivery. The assessment involved determining whether 6 prespecified essential elements were delivered: (1) provided advice on the health consequences of a high-salt diet, including recommended daily intake; (2) discussed the main sources of salt; (3) discussed and set goals to reduce salt intake; (4) discussed and set an action plan to achieve these goals; (5) delivered the SaltSwap leaflet; and (6) introduced and helped download the SaltSwap app. Following a discussion of discrepancies in the interpretation of the assessment framework, the remaining audio recordings were assessed by the principal investigator using the refined framework.

#### Secondary Outcomes

Secondary outcomes were the salt content of household food purchases recorded in the app (g/100 g), salt intake (g/day) estimated from 24-hour urinary sodium excretion, and blood pressure at 6-week follow-up. Although the nutrient of interest is sodium, nutrient labeling in the United Kingdom and public health messaging use the term *salt*. Therefore, the aim of this intervention was to encourage participants to reduce their *salt* intake, and the intervention highlighted the differences in the *salt* content of products not *sodium* content. Therefore, we measured the salt content, which was converted from urinary sodium excretion. For consistency, we referred to salt instead of sodium in this paper, unless referring to a study that has explicitly investigated sodium or when referring to urinary sodium excretion.

#### Process Outcomes

Process outcomes included recruitment rates; use and acceptability of the SaltSwap app; acceptability of the intervention by health care professionals, assessed through semistructured interviews; feasibility of outcome data collection; changes in participants’ knowledge about the effects of salt on health, use of nutrition labels for salt, and dietary salt behaviors, including the use of salt in cooking or at the table as well as consumption of high-salt foods; and contamination.

#### Qualitative Outcomes

Accompanied shopping and interview data were used in a qualitative analysis to explore the impact of the SaltSwap intervention on grocery shopping behavior, the use and acceptability of the SaltSwap app, and experiences of attempting to reduce salt intake.

### Statistical Analysis and Sample Size

Baseline characteristics were summarized using descriptive statistics by the trial arm. Statistical analyses were conducted using Stata IC 16.0 (StataCorp) [52]. Descriptive statistics were reported with 95% CIs. Progression criteria analysis used data from all available randomized participants. Secondary outcomes were analyzed on an intention-to-treat basis using a complete case analysis. Before calculating the salt content of purchased foods, missing data on product weight or volume or salt content were estimated where possible by cross-referencing web-based retailer websites; otherwise, products were excluded (considered *missing data*). Items categorized as fresh fruit and vegetables, alcohol, or household nonfood items were excluded from the analysis, as they included no or minimal salt.

We used linear regression models to calculate differences in means at follow-up between the intervention and control groups, with 95% CIs, adjusted for baseline values of the dependent variable and recruitment site. We examined the sensitivity of the results to confounding owing to differences in those baseline characteristics which were plausible confounders and imbalanced between groups. We also examined sensitivity of the results due to outliers. We tested the model fit using likelihood ratio tests. Where the assumptions of linear regression were not met, we applied the Wilcoxon rank-sum test to assess the outcomes. We also described the proportion of participants who showed a reduction in salt intake and purchase and assessed any differences in this proportion by trial group, using 2-tailed *t* tests for proportions.

Process measures used all data available, regardless of whether participants completed the trial, and were reported using descriptive statistics with 95% CIs. Qualitative data were analyzed using the Braun and Clarke [53] approach to thematic analysis. The lead author, an applied health services researcher specializing in public health interventions, analyzed the data with input from CA, a qualitative researcher with experience in thematic analysis. The coding framework was based on topics purposefully explored in interviews and those that developed from the data. Transcripts were coded line by line and then grouped into broad topics across cases. Interpretation of the data was informed by the COM-B model, the theoretical model of behavior change underpinning the intervention design [37]. Data quality and validity were enhanced by applying the techniques of Lincoln and Guba [54] to establish trustworthiness (Multimedia Appendix 4). Data were stored, coded, and managed using NVivo 12 (QSR International) for PC [55].

This study was a feasibility study and was not powered to detect any significant intervention effects. We calculated that a sample size of 40 would be sufficient to estimate progression outcomes within acceptably narrow CIs to enable robust testing of the trial methods. For the trial to be considered feasible, 80% follow-up attendance and fidelity of intervention delivery would need to be achieved. Therefore, allowing for a 95% CI around this point estimate, we set the minimum criteria for progression at 65%. For app use, a minimum of 70% was required; therefore, we set the minimum progression criteria at 50% based on the CI around this estimate. The think-aloud sample size of 12 was guided by the model of information power by Malterud et al [56], and sample selection was informed by recruitment site, participant gender, timing within the intervention period, and participant availability.

### Patient and Public Involvement

Before submission for ethical approval, we involved several members of an existing Patient and Public Involvement panel interested in weight loss and diet to inform and advise on the study objectives, procedures, and patient materials. We also convened a panel of members of the public with diagnosed hypertension to co-design the intervention.

### Adverse Events

All serious adverse events were reported during the trial. Participants were asked about adverse events at follow-up or at the point of withdrawal from the trial.

## Results

### Participants

**Figure 3:**
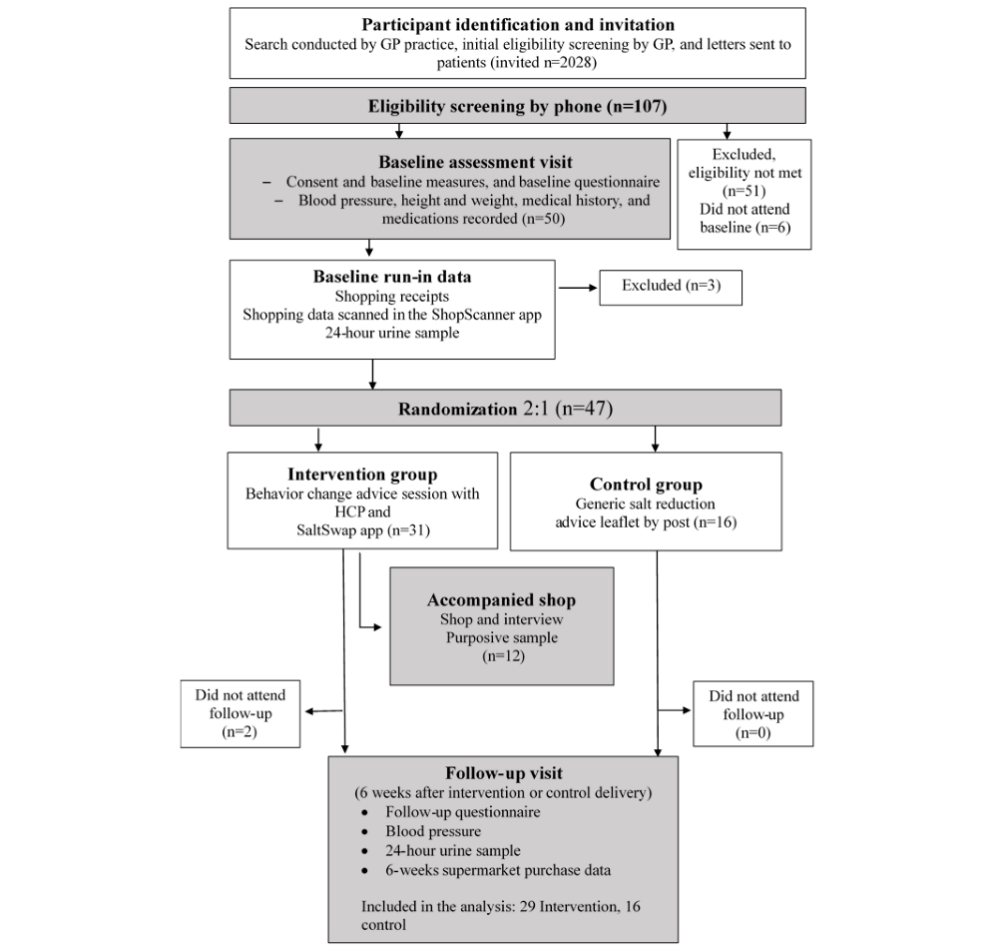
CONSORT (Consolidated Standards of Reporting Trials) diagram. GP: general practitioner; HCP: health care professional.

Participants were recruited between October 2018 and April 2019. A total of 2028 patients were deemed eligible based on blood pressure criteria and major exclusion criteria. Following a letter from the GP inviting participation in the trial, 107 patients registered their interest in taking part and completed phone-based screening with a researcher. Of the 107 patients, 51 (47.7%) did not meet the inclusion criteria. The main reasons for that were the following: not owning a smartphone (29/107, 27.1%), not being responsible for household grocery shopping (7/107, 6.6%), or being away during the study period (6/107, 5.7%). Out of the 56 patients who met the inclusion criteria, 6 (11%) did not attend the baseline assessment visit and 50 (89%) were enrolled (Figure 3). Participants had a mean age of 65 (SD 11) years, and the majority of the participants (35/47, 74%) reported a diagnosis of hypertension (Table 1). Only 13% (6/47) of all the participants had previously been advised by a health care professional to reduce their salt intake, although 48% (15/31) of the intervention group and 69% (11/16) of the controls reported that they had previously attempted to reduce their salt intake.

**Table 1:** Baseline characteristics of randomized study participants (N=47).

Characteristics		Control (n=16)	Intervention (n=31)	Total
Age (years), mean (SD)		67 (7)	64 (12)	65 (11)
Sex (female), n (%)		10 (63)	20 (65)	30 (64)
BMI (kg/m^2^), mean (SD)		29 (5)	29 (6)	29 (6)
**Blood pressure (mm Hg), mean (SD)**				
	Systolic	137 (15)	134 (16)	135 (15)
	Diastolic	80 (8)	81 (10)	81 (9)
Estimated daily salt intake^a^ (grams), mean (SD)		6.8 (2.7)	6.5 (3.9)	6.6 (3.5)
**Smoking, n (%)**				
	Current	0 (0)	1 (3)	1 (2)
	Ex-smoker	7 (44)	11 (35)	18 (38)
	Never	9 (56)	19 (61)	28 (60)
**Ethnic group, n (%)**				
	White	15 (94)	29 (94)	44 (94)
	Asian or Asian British	0 (0)	1 (3)	1 (2)
	Black or Black British	0 (0)	0 (0)	0 (0)
	Mixed or other or Chinese	1 (6)	1 (3)	2 (4)
**Education, n (%)**				
	No formal qualifications	4 (25)	0 (0)	4 (9)
	Secondary education	6 (38)	10 (32)	16 (34)
	Higher education	6 (38)	21 (68)	27 (57)
Household size, median (IQR)		2 (2-2)	2 (1-2)	2 (2-2)
**Weekly grocery shopping > £25 (US** $**34)/trip, n (%)**				
	More than once a week	5 (31)	12 (39)	17 (36)
	Once a week	9 (56)	15 (48)	24 (51)
	Once a fortnight	2 (13)	3 (10)	5 (11)
	Once a month	0 (0)	0 (0)	0 (0)
	Less than once a month	0 (0)	1 (3)	1 (2)
**Factors influencing grocery shopping decisions, n (%)^b^**				
	Price	6 (38)	20 (65)	26 (55)
	Appearance	0 (0)	5 (16)	5 (11)
	Taste	13 (81)	20 (65)	33 (70)
	Habits	5 (31)	7 (23)	12 (26)
	Health	9 (56)	17 (55)	26 (55)
	Convenience	1 (6)	5 (16)	6 (13)
	Special offers	7 (44)	8 (26)	15 (32)
	Organic	2 (13)	3 (10)	5 (11)
	Special diet	2 (13)	2 (6)	4 (9)
	Other	4 (25)	6 (19)	10 (21)
**Frequency of using nutrition labels, n (%)**				
	Salt	10 (63)	14 (45)	24 (51)
	Sugar	14 (88)	20 (65)	34 (72)
	Fat (total or saturated)	13 (81)	21 (68)	34 (72)
	Energy (calories)	13 (81)	21 (68)	34 (72)
Been advised by a health care professional to reduce their salt intake, n (%)		2 (13)	4 (13)	6 (13)
Previously tried to reduce salt intake, n (%)		11 (69)	15 (48)	26 (55)
**Knowledge of the effect of salt on blood pressure?, n (%)^c^**				
	Yes	8 (50)	11 (35)	19 (40)
	No	1 (6)	7 (23)	8 (17)
	Do not know	7 (44)	13 (42)	20 (43)
Eating breakfast out, median (IQR)		0 (0-0.5)	0 (0-0)	0 (0-0)
Eating lunch out, median (IQR)		0 (0-1)	1 (0-2)	1 (0-1)
Eating dinner out, median (IQR)		1 (0-1)	0.5 (0-1)	1 (0-1)
**Relevant health history, n (%)**				
	CVD^d^	0 (0)	4 (13)	4 (9)
	Diagnosed hypertension	14 (88)	21 (68)	35 (74)
	Diabetes	4 (16)	3 (10)	7 (15)
	Atrial fibrillation	0 (0)	0 (0)	0 (0)
	Chronic kidney disease	0 (0)	1 (3)	1 (2)
	Peripheral vascular disease	1 (7)	1 (3)	2 (4)
	Other (related to CVD)	5 (31)	5 (16)	10 (21)
	Antihypertensive medication	12 (75)	19 (61)	30 (64)

^a^Converted from 24-hour urinary sodium excretion.

^b^Participants were asked to choose the top three factors.

^c^Participants were asked, “Do you think the amount of salt you eat affects your blood pressure?”

^d^CVD: cardiovascular disease.

### Primary Outcomes

All 3 progression criteria were met well above the set thresholds (Table 2). The total attendance at follow-up was 96% (45/47; 29/31, 94% in the intervention group and 16/16, 100% in the control group). There were 2 participants who did not attend follow-up; 1 withdrew because of difficulty using the study app and 1 because of an unrelated health problem. Of the intervention sessions that were adequately recorded (25/31, 81%), all included at least 4 of the 6 essential elements. All 6 elements were delivered in 21 (84%) recorded sessions; the element most commonly not delivered was action planning (3/25). A total of 27 out of 31 (87%) intervention participants used the SaltSwap app to scan products on at least 1 occasion by the end of month 1, above the progression threshold of 50%.

**Table 2:** Participant follow-up rate, fidelity of intervention delivery, and use of the SaltSwap app (n=47).

Progression criteria	Total			Control (n=16)			SaltSwap (n=31)	
	n (%)	95% CI	n (%)		95% CI	n (%)		95% CI
Follow-up rate	45 (96)	85-99	16 (100)		79-100	29 (94)		79-99
Fidelity of the intervention (advice) session^a^	N/A^b^	N/A	N/A		N/A	25 (81)		63-93
Use of the SaltSwap app	N/A	N/A	N/A		N/A	27 (87)		70-96

^a^Audio recordings for the assessment of intervention fidelity were available for 25 of the 31 advice sessions delivered.

^b^N/A: not applicable.

### Secondary Outcomes

Baseline and follow-up data on the salt content of the purchased products were available for 78% (37/47) of participants. The analysis included all the purchased items recorded in the app. The number of products purchased was slightly lower at follow-up in both groups, and missing data were comparable between groups (Multimedia Appendix 5).

**Figure 4:**
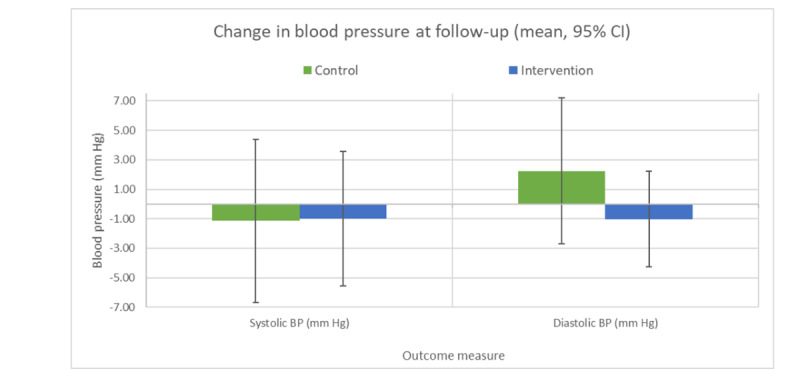
Changes in blood pressure from baseline to follow-up. BP: blood pressure.

There was no evidence that the intervention significantly reduced the salt content of purchased foods, salt intake, or blood pressure, and there was no significant between-group difference (Table 3; Figure 4). In the intervention group, 72% (18/25) of the participants reduced the salt content of purchased foods (mean −0.15 g/100 g, SD 0.13 g/100 g) compared with 66% (8/12) of the control group (mean −0.4 g/100 g, SD 0.27 g/100 g). Similarly, 50% (14/28) of intervention participants reduced their salt intake from baseline to follow-up (mean reduction −2.44 g/day; 95% CI −8.71 to −0.20 g/day) compared with 66% (10/15) of the control group (mean reduction −2.42 g/day; 95% CI −6.76 to −0.08 g/day). The differences in these percentages were not statistically significant. There were nonsignificant reductions in expenditure on food at follow-up for both groups, with no significant between-group differences.

**Table 3:** Changes in the mean salt intake, blood pressure, and purchased salt from baseline and estimates of differences between the intervention and control groups.

				Baseline values, mean (SD)				Follow-up values, mean (SD)				Change, mean (95% CI)					Group difference^a^, mean (95% CI)			*P* value
				Control		SaltSwap		Control		SaltSwap		Control		SaltSwap		SaltSwap vs control				
**Salt intake (g/day)**				6.8 (2.7)		6.5 (3.9)		6.0 (2.1)		6.2 (3.7)		−1.0 (−2.4 to 0.4)		−0.2 (−1.4 to 0.9)		−0.4 (−2.3 to 1.5)			.68	
	Number of participants, n		16		31		15		28		15		28		43			N/A^c^		
**BP^b^ (mm Hg)**																				
	Systolic BP		137.3 (14.8)		134.2 (15.8)		136.2 (15.9)		133.2 (16.9)		−1.1 (−6.7 to 4.4)		−1.0 (−5.5 to 3.6)		N/A			.82^d^		
	Diastolic BP		79.7 (8.0)		81.2 (10.2)		82.0 (10.5)		80.2 (10.7)		2.3 (−2.7 to 7.2)		−1.0 (−4.3 to 2.2)		−3.0 (−8.0 to 2.0)			.23		
	Number of participants, n		16		31		16		29		16		29		45			N/A		
**Purchased salt (g/100g)**				0.8 (0.5)		0.5 (0.2)		0.6 (0.4)		0.5 (0.4)		−0.1 (−0.5 to 0.2)		0.0 (−0.1 to 0.2)		N/A			.16^d^	
		Number of participants, n	12		25		12		25		12		25		N/A			N/A		

^a^Model 2. Regression analysis adjusted for baseline values, general practitioner surgery, and level of education.

^b^BP: blood pressure.

^c^N/A: not applicable.

^d^*P* value for the Wilcoxon rank-sum test for between-group differences. Assumptions of linear regression were not met for systolic blood pressure and salt content of purchased foods; therefore, we analyzed these outcomes using the Wilcoxon rank-sum test.

### Process Outcomes

The intervention was acceptable to both individuals and practitioners, and outcome data were successfully collected via trial methods. The SaltSwap app was used (ie, at least one product scanned) in 75% of all shopping trips by intervention participants, and 79% (23/29) of intervention participants who used the app recorded at least one swap to a lower-salt product in the app. The average salt reduction per swap recorded in the app was −0.6 g/100 g. Participants in both groups reported eating high-salt foods less frequently and swapping to lower-salt foods (Multimedia Appendix 5).

### Qualitative Outcomes

A total of 11 intervention participants attended an accompanied shopping trip and postshopping interview, and 1 participant attended only a postshopping interview (Multimedia Appendix 4). Overall, the qualitative study sample reflected the demographic profile of the total sample. The average age of the participants in this substudy was 67 (SD 7) years compared with 65 (SD 11) years in the total sample, representative of the age of the population with hypertension. The qualitative study comprised 58% (7/12) female participants compared with 64% (30/47) in the total sample, and all but 2 of the qualitative participants were of White British ethnicity, reflecting the lack of ethnic diversity in the overall study sample. Shopping trips varied in duration from 10 to 44 minutes and interviews from 17 to 40 minutes, with an average combined duration of 48 minutes per person. Key themes were developed around 3 main areas: SaltSwap intervention, salt consumption and purchase behaviors, and the wider environment. Detailed theme descriptions and example quotes are presented in the Multimedia Appendix 4.

Participants reported improved knowledge about the major sources of salt and heightened awareness of the importance of salt intake in relation to blood pressure. They described greater attention to the salt content of foods than before the study, showing surprise at the high salt content of some foods they routinely buy and at the availability of lower-salt options. All these aspects were explicitly targeted by the intervention:

...doing this it's heightened my awareness of salt in the diet...I was horrified at the levels of salt in crisps and things, so have gone for the no adding salt to that. Sauces, I've been surprised how much salt is in those. [ID0205; female, 68 years]

The advice session helped participants identify specific high-salt foods to swap and successfully encouraged them to set goals to make changes to their purchases. There was little evidence of action planning during the intervention session or mention of doing this afterward. Interview data suggested that the intervention did not sufficiently generate motivation to reduce salt intake. Some participants indicated that they would be more motivated if they received feedback on their salt intake from baseline 24-hour urine measurement:

I guess I'd like to know where I am on the spectrum. So, as a result of the urine tests. Where do I sit...in terms of a male of my age? Because that may provide something...motivation. [ID0309; male, 71 years]

All intervention participants were observed or described using the SaltSwap app to identify lower-salt alternatives with the app. Most participants found the app usable and acceptable and noted that it helped focus their attention on the salt content of foods. Few participants reported using the feedback function in the app, showing a reduction in the salt content of selected items through making swaps.

Barriers to successfully identifying lower-salt products and continued use of the app were the lack of comprehensive coverage of products within the app database, unsuitable suggestions for lower-salt alternatives, and lack of availability of suitable lower-salt options in some stores or for specific food groups:

This is...tomato paste. See, I think being [store] they only do one. [ID0206; male, 64 years]

Participants reported changing their shopping behaviors, for example, swapping to lower-salt foods, avoiding buying high-salt foods entirely, and changing their cooking practices to reduce their salt intake (Table 4). This sometimes resulted in participants avoiding or reducing the frequency of purchasing foods, which were also higher in other nutrients associated with poor diet quality, such as saturated fat. Participants reported increasing their use of nutrition labels for salt following the intervention, often in conjunction with the SaltSwap app, although some participants still reported challenges interpreting nutrition labels:

I would say since I've started the salt study, I've looked at those labels more than in the past. In the past I used to only look at the sugar content. [ID0403; male, 70 years]

**Table 4:** Examples of participants-reported behavior change and the related COM-B components (intervention participants).

Behaviors	Example quotes	COM-B components
Swapping to a lower-salt product	“Now we used to buy the deals here of different sorts of ham – but when I tried to do a salt swap on them it was quite difficult to find an alternative. So, what we do now is we buy sliced turkey” [ID0403; male, 70 years] “I used to buy crisps without thinking about salt. And then I started the salt file, I just switched to these.” [ID0313; female, 57 years] “I used to buy a tomato sort of sauce. And in fact, because that was quite high in salt, I started using Passata little boxes. Because you get the sort of intense tomato flavour, but you don’t get the salt.” [ID0410; female, 74 years] “Like I haven't been buying so much ham. I have bought chicken instead.” [ID0415; female, 71 years]	Psychological capability—knowledge Reflective motivation Automatic motivation—disrupting habit Environmental opportunity—making it easy to identify lower-salt options
Avoiding high-salt foods	“They were like a ripple type crisp, and I was surprised how much salt was in that. And in fact, I don’t think I bought them in the end. Because I thought, 'Wow, I thought these were supposed to be, you know, healthy.” [ID0206; male, 64 years] “Anyway, I used to buy those particularly when it was the two for one type offers. Just stopped buying those completely, yeh” [ID0408; male, 65 years] “I do like salted peanuts and I haven't been able to find anything that’s low in salt...I probably would have bought them most weeks. Whereas now I think, ‘No, it is a treat.’... I won't have those.” [ID0205; female, 68 years]	Psychological capability—knowledge Reflective motivation—intentions or persuasion Automatic motivation—disrupting habit
Changing cooking practices to reduce salt intake	“And I've also...we've also this last week haven't cooked with salt either. Yeh, previously if we used in cooking potatoes; boiling potatoes up for mashed potatoes; always put a bit of salt in there. Even with your, you know, vegetables, put a bit of salt in there, but we haven't this time.” [ID0207; male, 58 years] “So, instead of using stock cubes. Which we know are high in salt, I tend to use Bouillon because I can use just a tiny bit of that. I will sometimes use that. Or what I do is I add that after I've taken my portion. And that, because it's not a stock cube has to be whatever, and what have you. With that I can stir that in...So, that works quite well.” [ID0319; female, 69 years]	Psychological capability—knowledge and skills Automatic motivation—disrupting habit
Swapping from a store-bought product to homemade, with no added salt	“After I'd seen those things and how high they were in salt, when I went and did my own, I didn’t put any salt whatsoever in it at all, and I just used the Indian spices” [ID0218; female, 76 years]	Psychological capability—knowledge and skills Reflective motivation—persuasion
Reducing frequency of consumption or portion size	“I probably would have bought them most weeks. Whereas now I think, ‘No, it is a treat.’ And I would also not sit and eat the whole packet.” [ID0205; female, 68 years]	Psychological capability—knowledge Reflective motivation—intentions

The time and effort required to find lower-salt alternatives was commonly mentioned as a barrier to changing their purchasing behavior, but others described making new habits and planning their shopping as ways to help make and stick to lower-salt choices.

Some participants found it challenging to balance salt reduction with other nutritional concerns, such as saturated fat or sugar. They also expressed a desire for feedback on the outcome of their behavioral change, that is, explicit measurement of their salt intake, highlighting the potentially important role of feedback in increasing motivation.

Participants commented on the choice of products available, both that there was too much choice to make it easy to choose low-salt options and that some stores do not offer lower-salt options. They generally felt that there was a lack of support in making dietary changes to reduce their salt intake but that their GP surgery would be a good place to provide support of this nature.

### Adverse Events

One serious adverse event was reported during the study. The lead GP for the surgery concluded that this adverse event was unrelated to the study. The participant did not continue in the study or attend the follow-up, but no further action was taken.

## Discussion

### Principal Findings

This trial intended to assess the feasibility of the SaltSwap intervention and trial procedures to determine if a larger trial to assess the effectiveness of the intervention for reducing blood pressure is warranted. The results showed high levels of follow-up, fidelity of intervention delivery, and use of the intervention app, demonstrating that progression criteria were met. Health care professionals were positive about the intervention and confident in delivering it, indicating that the SaltSwap intervention can be delivered by nonspecialist staff in primary care without intensive training. Furthermore, intervention participants used the app in store in most shopping trips, and the majority used the app to identify and select lower-salt alternatives, demonstrating high acceptability among individuals with high blood pressure. The app product database provided insufficient coverage of in-store products, and product categorization resulted in some unsuitable lower-salt suggestions, hindering effective use.

Although there were no significant effects on purchased salt, salt intake, or blood pressure, CIs around each point estimate included clinically meaningful reductions, and the intervention was successful in changing key salt reduction behaviors, suggesting that the intervention could be potentially successful if tested in a larger trial. The trial procedures to evaluate the effect of the intervention on salt intake and blood pressure were shown to be robust.

### Strengths and Limitations

A key strength of this study is the in-depth qualitative analysis and process evaluation to assess the proposed *mechanisms of impact* of this novel intervention, providing insight into context-specific use of the app and barriers to the intervention’s potential effectiveness. Participants described increased knowledge, intention to reduce salt intake, and ability to identify lower-salt options—key targets of the intervention based on our COM-B behavioral analysis. Process outcomes demonstrated that participants swapped to lower-salt items predominantly in the food categories of bread, cheese, and processed meats, foods that contribute most to total daily salt intake (accounting for 34%) among those aged 65-74 years [57], suggesting that the intervention successfully targeted the main sources of dietary salt.

Another strength is the development and proven feasibility of a behaviorally informed smartphone app tailored to the UK grocery market, which can support individuals to make lower-salt choices when grocery shopping and provide feedback on salt reduction. Smartphone use in the United Kingdom is ubiquitous and has grown dramatically; a 2019 report found that most adults in the United Kingdom use a smartphone phone (84%), and this is true for all age groups, including 93% of 35- to 54-year-olds and 64% of those aged ≥55 years [58]. However, the use of mobile health apps is not as widespread among older adults [59], and app-based interventions may still present specific age-related barriers to the effective use of smartphone apps [60]. This is an important concern when addressing a population with hypertension, including many older adults [61]. This trial successfully engaged a population of adults with an average age of 64 years with a mobile app–based intervention with few usability issues, demonstrating the feasibility of delivering app-based health behavior change interventions to older adults. A previous study investigating barriers to app use in older adults (aged >60 years) found the main barriers to be concerns about data privacy, lack of trust, and fear of misdiagnosis [59]. The support of a trusted health care professional in the use of the app could potentially overcome barriers of this nature. Moreover, as the app does not require any personal data or any health care data and does not communicate information about any individual to any third party, these common barriers are unlikely to be significant concerns for SaltSwap. Health care professionals successfully delivered the intervention advice session. Although audio recording of the intervention may have acted as a prompt to deliver the advice elements, introducing a Hawthorne effect [62], the audio recordings provided evidence that the intervention content was within their expertise and skill set to deliver competently.

The trial response rate of only 5% was a limitation. Although comparable with other trials of a similar nature [35,63], it is lower than that for other dietary intervention trials, such as those targeting weight loss [64]. The low response rate raises two issues. First, it may indicate less interest in reducing salt intake than other dietary goals, presenting a challenge for the implementation of the intervention. In clinical practice, brief interventions are likely to be given in consultation, regardless of patients’ inclination to reduce salt intake or interest in using an app. Such respondents may differ from those we enrolled, meaning that the results here, indicating the acceptability of the intervention and potential for changing salt intake behaviors, may not generalize to the wider population of people with hypertension.

Second, the low response rate may indicate that this trial engaged a group of individuals with high agency and motivation to address their diet. This intrinsic motivation could lead to participants actively engaging with the control intervention (self-help booklet) and may explain why salt intake reduced in both arms, although not significantly, reducing the apparent impact of the SaltSwap intervention. Indeed, salt intake at baseline was lower than the age-adjusted national average, and many participants reported previous attempts to eat less salt. In addition, measurement reactivity may have resulted in participants being particularly careful to reduce salt intake on measurement days, biasing results toward the null. A larger effectiveness trial would do well to exclude people with low baseline salt intake and to consider multiple measurements at both baseline and follow-up, including both weekdays and weekends, to ensure a more representative salt intake assessment. Furthermore, urine samples could be used to assess potassium levels in a future trial to explore any salt substitution effects.

The study population was self-selected—for reasons of convenience, drawn solely from Oxfordshire, an area with lower deprivation than the national average [65]. Socioeconomic status is known to influence salt intake and adherence to wider dietary guidelines [66-68], and the impact in this group may not be representative of the wider population. Moreover, the study participants were predominantly of White ethnicity. Salt intake varies across ethnic groups [69,70], and culture is a major determinant of food choice and consumption [70,71]. However, the database includes a wide range of foods available in supermarkets and should cover most dietary preferences, making the app universally applicable. The literature on the differential impacts of salt reduction interventions across ethnic groups is sparse, and there is a lack of evidence on specific barriers and facilitators for food purchasing and dietary behavior change among minority ethnic groups to adequately inform intervention adaption [72]. An important consideration for any future trial of the SaltSwap intervention is the recruitment of a study sample with greater ethnic and socioeconomic diversity.

A further limitation was the lack of comprehensive product coverage within the SaltSwap app, despite it containing more than 95,000 unique products. Participants noted this as a key barrier to successfully identifying lower-salt options in stores, which may have affected the resulting salt reduction. This also resulted in a high level of missing data on the salt content of purchased foods, thus limiting the interpretation of this secondary outcome; however, this is unlikely to have biased the outcome differently by arm [73]. Participants used the app as asked; therefore, an improved database would reduce missing purchase data in future trials. Data on other important dietary components that may be affected by this intervention were also not captured through the SaltSwap app; however, with improvements to the app database, these could be included as secondary outcomes in a larger trial.

### Comparison With Other Literature

There are few RCTs of the impact of apps alone or combined with other interventions to reduce salt intake [74], but the available evidence suggests that this is a promising strategy. One trial showed that a standalone app to encourage the purchase of lower-salt foods reduced salt purchasing but not salt intake or blood pressure [34]. Another RCT in a small sample of healthy adults showed short-term reductions in sodium consumption in participants using an app to record and monitor their daily sodium intake [33]. A recent pilot study of an app using geolocation to prompt users to choose lower-sodium options when shopping or eating out or at home showed no significant reduction in 24-hour sodium compared with usual care and no change in confidence adhering to a low-sodium diet (including reading food labels, shopping at grocery stores, or choosing low-sodium options at restaurants) but did show a significant reduction in spot urine as an estimate of sodium intake and sodium intake measured by a food frequency questionnaire [35]. These trials all investigated standalone app interventions as compared with the multicomponent nature of SaltSwap. A review of behavior change apps for dietary change highlighted that multicomponent interventions had, on average, larger effect sizes than single-component interventions [32]. Furthermore, existing evidence shows that brief behavioral support from a health care professional can generate the necessary capability and motivation to make dietary changes [28-30], which could be supported by an app at the point of choice. The theoretical approach to intervention development highlighted the need for a salient and persuasive message to motivate individuals to change their diet, and SaltSwap aimed to deliver this via in-person advice from a credible source. We found no other studies in the literature of a multicomponent intervention using an app to enhance a brief intervention for salt reduction; therefore, this feasibility study provides valuable insight into the feasibility and acceptability of such an approach.

For the SaltSwap intervention to have a meaningful effect on blood pressure, it would need to generate a reduction in salt intake of at least 1 to 2 g/day. A meta-analysis of RCTs investigating the effect of salt reduction on blood pressure reported an average reduction in salt intake of −4.6 g/day, which led to reductions in systolic (5.0 mm Hg) and diastolic blood pressure (2.7 mm Hg) [75]. However, a later RCT showed that a low-salt diet resulted in a modest salt reduction of 1.7 g/day, with a corresponding reduction in systolic blood pressure of 3.7 mm Hg, among participants with high blood pressure [76], indicating the potential for clinically meaningful benefit from a more modest salt reduction. In fact, individual counseling interventions for salt reduction, delivered by specialist dieticians, have been shown to be cost-effective based on expected reductions in salt intake of 0.5 g/day [77]. A reduction of 1 to 2 g/day falls within the CI for the salt reduction achieved here, in a sample of individuals with comparatively low baseline salt intake, who may have a limited capacity for further reduction. The magnitude of change in objectively measured salt intake and blood pressure, along with the behavioral changes identified in our qualitative evaluation, provides a sufficient signal of efficacy to warrant progression to a full trial.

In the United Kingdom, as with many other high-income countries, where most of the salt consumed is already in the food chain and the food environment promotes high-salt, highly processed foods, providing advice, knowledge, and motivation is unlikely to be enough on its own to provide sufficient reduction in salt intake to achieve national recommendations. Concurrent structural interventions, such as product reformulation and nutrition labeling, are important salt reduction strategies, creating an environment conducive to individual-level behavior change [78]. For people to habitually choose lower-salt options, these need to be readily available, acceptable, and identifiable without undue effort. There is a synergistic relationship between individual-level and structural, population-level interventions. In the United Kingdom, a voluntary reformulation strategy has resulted in significant reductions in the mean salt content of many food categories that contribute substantially to salt intake (eg, cereals, breads, and snack foods) [79]. However, one result of this approach is that some manufacturers have made ambitious progress, whereas others have lagged behind, leading to a wide variation in salt content across some food categories [80,81]. With such a variation in salt content across foods, the full benefit of the reformulation strategy can only be achieved when individuals are motivated and enabled to choose available lower-salt options, reinforcing the need for individual-level interventions that can support people to do this. This, in turn, could give companies that reduce salt in their products a competitive edge, stimulating a virtuous cycle of salt reduction.

### Conclusions

A behaviorally informed, brief intervention to reduce salt intake in people with hypertension, delivered in primary care and supported by an app to inform purchasing decisions, was acceptable to those delivering and those receiving it, and the trial approaches are considered feasible. A full trial to assess the effectiveness of the SaltSwap intervention for reducing salt intake and blood pressure is warranted following improvements to the SaltSwap app highlighted as important during this feasibility study.

## Appendix

Multimedia Appendix 1 Intervention development.

Multimedia Appendix 2 The SaltSwap app description and technical information.

Multimedia Appendix 3 The ShopScanner app.

Multimedia Appendix 4 Qualitative methods and outcomes.

Multimedia Appendix 5 Process outcomes.

Multimedia Appendix 6 CONSORT-eHEALTH checklist (V 1.6.2).

## Abbreviations

BCT: behavior change technique
CVD: cardiovascular disease
GP: general practitioner
NIHR: National Institute for Health Research
RCT: randomized controlled trial
