# Supplementary material for: A Mobile Health Salt Reduction Intervention for People With Hypertension: Results of a Feasibility Randomized Controlled Trial
Source: JMIR Mhealth Uhealth. 2021 Oct 21;9(10):e26233. doi: 10.2196/26233 (PMC8569539; doi:10.2196/26233)
Supplement: Multimedia Appendix 1 [file mhealth_v9i10e26233_app1.docx]

# Appendix 1: Intervention development

We designed the SaltSwap intervention, using the Behaviour Change Wheel.

We identified the target behaviour as *reducing purchases of high-salt foods*, based on the major contribution of processed food to salt intake. We used the COM-B model to identify aspects of capability, motivation and opportunity which needed to change to encourage this behaviour (Supplementary Table 1). The behavioural diagnosis concluded that key elements of psychological capability, social and physical opportunity and both reflective and automatic motivation needed to be addressed to reduce the purchase of high salt foods. This informed key intervention objectives as increasing knowledge around salt intake and health, enhancing intrinsic motivation to change, increasing self-efficacy to reduce their salt intake, and improving their opportunity to do so. We selected 16 behaviour change techniques (BCTs) (Supplementary Table 2), from The Behaviour Change Technique Taxonomy [38]. SaltSwap is a multi-component intervention including a smartphone app and a face-to-face brief advice session with a healthcare practitioner; the app enables delivery of BCTs which could not easily be delivered through a single face-to-face session e.g. tailored advice on purchases, feedback on the outcome of behaviour, and the advice session provides verbal persuasion and problem-solving, which could less easily be delivered by a stand-alone app intervention. The face-to-face advice session provides support to participants to start using the app, which in an older population likely to have less experience with apps lower app-use, may be a key element in achieving sufficient app engagement. The SaltSwap app was developed by a third-party software developer and version 1.0.6 was evaluated in this trial, with no version change of updates during the intervention. (Appendix 2).

Supplementary Table 1. COM-B components, TDF domains and what needs to change to reduce individuals’ purchase of high-salt foods

| **COM-B component** | **TDF domain(s)** | **What needs to change for the target behaviour to occur?** |
| --- | --- | --- |
| Physical Capability | Physical Skills | No changes required. |
| Psychological capability | Knowledge  Cognitive & interpersonal skills  Memory, attention & decision making  Behavioural regulation | Increase knowledge about why to reduce salt; the health consequences of high salt intake and the benefits of reducing it; knowledge of how much salt is recommended; what the most common sources of salt in the diet are; and how to identify high-salt foods and lower-salt alternatives. Knowledge and understanding of their personal salt intake.  Improve skills for using nutrition information on packs  Improving decision-making between alternative products  Enhance self-monitoring of progress towards goals e.g. facilitate feedback and encourage action planning to achieve a pre-selected goal. |
| Physical opportunity | Environmental contextual & resources | Changes to the environmental context to make lower-salt options more easily identifiable |
| Social opportunity | Social influences | Improve family support for lowering salt intake  Manipulation and communication of social norms towards purchase of lower-salt products |
| Reflective motivation | Goals  Intentions  Beliefs about consequences  Optimism  Beliefs about capabilities Professional/social role & identity | Increase intention to reduce salt intake, particularly through purchasing lower-salt alternatives, and fewer high-salt products  Persuade people that reducing their salt intake really could improve their health and blood pressure  Increase peoples’ belief in their capability to reduce their salt intake  Encourage and facilitate goal setting to reduce salt intake  Encourage people to see eating a low-salt diet as part of their identity as someone who lives a healthy lifestyle |
| Automatic motivation | Reinforcement  Emotion | Disrupt automatic purchases of ‘habitual’ products |

| Supplementary Table 2. BCTs delivered through the intervention to reduce individuals purchase of high-salt foods, and the selected mode of delivery | | | |
| --- | --- | --- | --- |
| **BCT** | **Definition** | **Delivered in SaltSwap** | **Mode of Delivery** |
| 1.1 Goal Setting | Set or agree on a goal defined in terms of the behaviour to be achieved | Set a goal to make swap a set number of products to lower-salt alternatives | Face-to-face  App |
| 1.2 Problem Solving | Analyse, or prompt the person to analyse, factors influencing the behaviour and generate or select strategies that include overcoming barriers and/or increasing facilitators | Discuss the individuals’ perceived barriers to swapping to lower-salt alternatives and how to overcome these. | Face-to-face |
| 1.4 Action planning | Prompt detailed planning of performance of the behaviour (must include at least one of context, frequency, duration and intensity). Context may be environmental (physical or social) or internal (physical, emotional or cognitive) | Complete an action plan detailing which products to find alternatives for (each week), and where and when they will do this. | Face-to-face |
| 2.2 Feedback on Behaviour | Monitor and provide informative or evaluative feedback on performance of the behaviour | Feedback provided on the number of swaps to lower-salt alternatives accepted each shopping trip, in relation to their goal | App |
| 2.3 Self-monitoring of behaviour | Establish a method for the person to monitor and record their behaviour(s) as part of a behaviour change strategy | Show the number of swaps made, and what they were, for each shopping trip | App |
| 2.4 Self-monitoring of outcome of behaviour | Establish a method for the person to monitor and record the outcome(s) of their behaviour as part of a behaviour change strategy | Show the percentage reduction in salt achieved through the swaps made, for each shopping trip | App |
| 2.7 Feedback on Outcome of Behaviour | Monitor and provide feedback on the outcome of performance of the behaviour | Provide feedback on the percentage salt reduction achieved through swaps made, for each shopping trip | App |
| 4.1 Instruction to perform behaviour | Advise or agree on how to perform the behaviour | Show the person how to identify lower-salt options, using the app and product nutrition labelling and also explain other ways to reduce salt intake | Face-to-face |
| 5.1 Information about health consequences | Provide information (e.g. written, verbal, visual) about health consequences of performing the behaviour | Explain the effect of reducing salt intake on blood pressure and risk of CVD | Face-to-face |
| 7.1 Prompts/cues | Introduce or define environmental or social stimulus with the purpose of prompting or cueing the behaviour. The prompt or cue would normally occur at the time or place of performance | Use of the SaltSwap app as an environmental stimulus to look for salt content of products or for products labelled ‘reduced-salt’. The traffic light labels in the app will also be prompts to change to a lower-salt product | App |
| 8.2 Behaviour substitution | Prompt substitution of the unwanted behaviour with a wanted or neutral behaviour | Provide suggested alternatives to high-salt products | App |
| 9.1 Credible source | Present verbal or visual communication from a credible source in favour of or against the behaviour | Salt reduction advice presented by a trained healthcare professional. Include references to evidence in the scientific literature to support the benefits of salt reduction. | Face-to-face |
| 11.3 Conserving mental resources | Advise on ways of minimising demands on mental resources to facilitate behaviour change | Use of the app to make it easier to identify lower-salt alternatives, as the best alternative is shown | App |
| 12.5 Adding objects to the environment | Add objects to the environment in order to facilitate performance of the behaviour | Via the app, add traffic light nutrient labels and suggested lower-salt alternatives, into the shopping environment to aid identification of lower-salt products | App |
| 13.1 Identification of self as a role model | Inform that one's own behaviour may be an example to others | Encourage the person to share their successes in finding lower-salt options with their friends and family | App |
| 15.1 Verbal persuasion about capability | Tell the person that they can successfully perform the wanted behaviour, arguing against self-doubts and asserting that they can and will succeed. | Discuss the person’s capability to swap to lower-salt alternatives and stick with them. Demonstrate that they can successfully reduce their salt intake without giving up all their favourite foods. | Face-to-face |
