# Supplementary material for: A Mobile Health Salt Reduction Intervention for People With Hypertension: Results of a Feasibility Randomized Controlled Trial
Source: JMIR Mhealth Uhealth. 2021 Oct 21;9(10):e26233. doi: 10.2196/26233 (PMC8569539; doi:10.2196/26233)
Supplement: Multimedia Appendix 2 [file mhealth_v9i10e26233_app2.docx]

# Appendix 2. SaltSwap app description and technical information

## Access

The app was free to use for participants, who downloaded the app from the Apple App store (for iPhone) or Google Play (for Android) and created a user account and login themselves.

## About SaltSwap

The SaltSwap App has been developed by the University of Oxford, funded by the British Heart Foundation and the National Institute for Health Research. The app aims to help you to identify the amount of salt in everyday packaged foods and to reduce the amount of salt you eat by swapping to lower salt alternatives.

**How it works**

When you scan the barcode of a product, the app displays the nutrition information for packaged foods, using a colour coded traffic light nutrition label. This label shows the amount of salt in the food or drink, colour coded as **RED** for high salt, **AMBER** for moderate and **GREEN** for low. The nutrition information is provided as grams of salt per 100g of the food (or per 100mls for drinks). Try to avoid foods which show **RED** for salt by swapping to a lower salt alternative or eat them less frequently as this will also help you reduce your salt intake. The label also shows total fat, saturated fat, sugar and energy.

The app also displays how much less salt there is in the alternative product compared to the one you scanned – in the blue circle. This is also shown in grams of salt per 100grams.

For each item you scan you can choose to add it to your shopping basket or to swap to one of the alternatives shown. At the end of your shopping trip the app will calculate how much you have reduced the salt in your total shopping basket through the swaps you have made, shown as a percentage reduction in salt.

You can also search directly for products without scanning them.

**Where does the nutrition information come from?**

The nutrition information is sourced from large product databases which contain most commonly bought packaged food and drinks – gathered through real product barcodes. We rely on manufacturers providing this nutrient information and, in some cases, the full nutrition information is not available. We don’t make estimates for missing nutrition information, the app will show this as n/a.

**How does the app find alternatives?**

The app will show you a selection of similar products with at least 10% less salt per 100 grams. It may also show you some ‘less similar’ alternatives which have a larger reduction in salt as these can really help you to reduce your daily salt intake. You can find other alternatives by scanning similar products from the grocery shelf. As this app is focused on reducing the amount of salt you eat, some alternative products might be a bit higher in sugar or fat. You can check the colour coded nutrition information for this.

Product and nutrition data for this app was provided by The George Institute for Global Health, for research purposes. The SaltSwap app is no longer publicly available however a newer version is being developed as part of ongoing research. To enable a degree of digital preservation of the intervention app, screen shots of the SaltSwap app are included here

**Instruction screens 1-3**


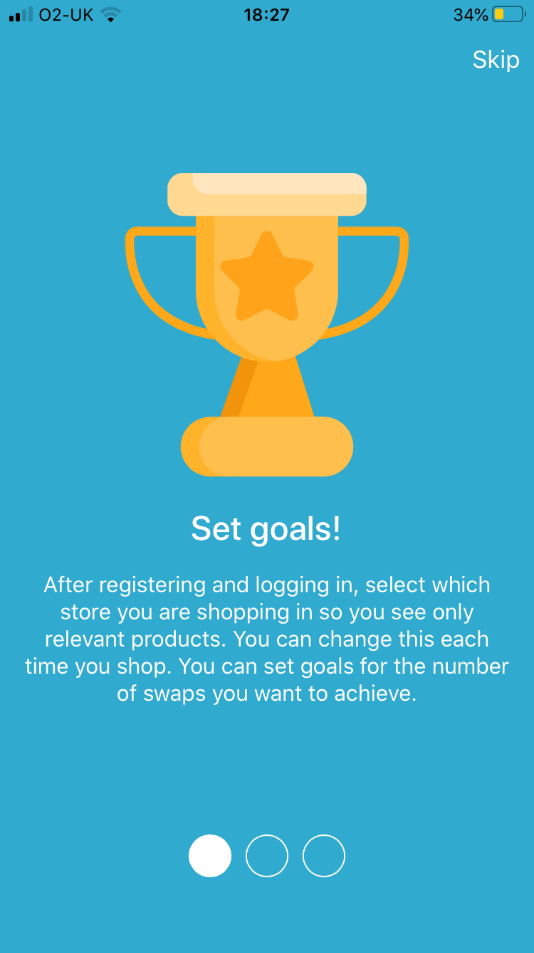

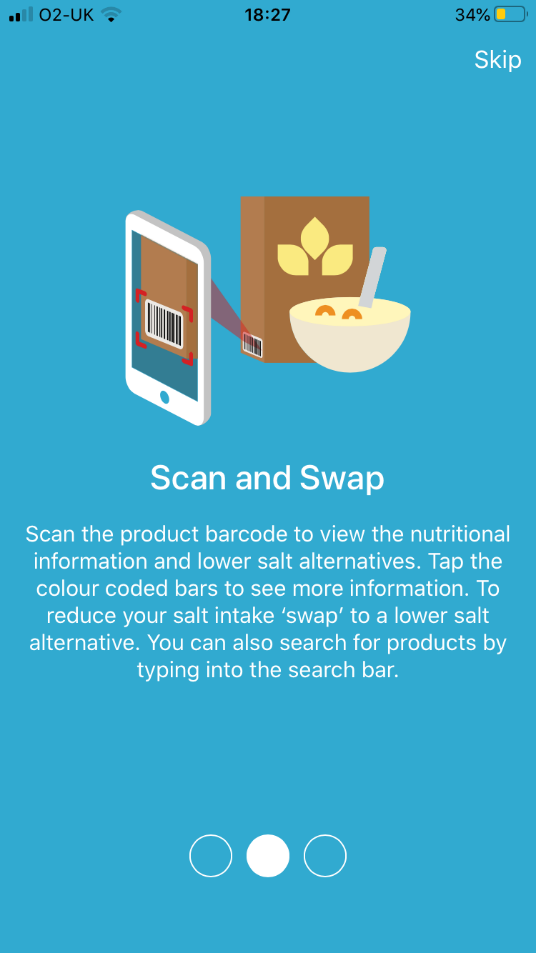

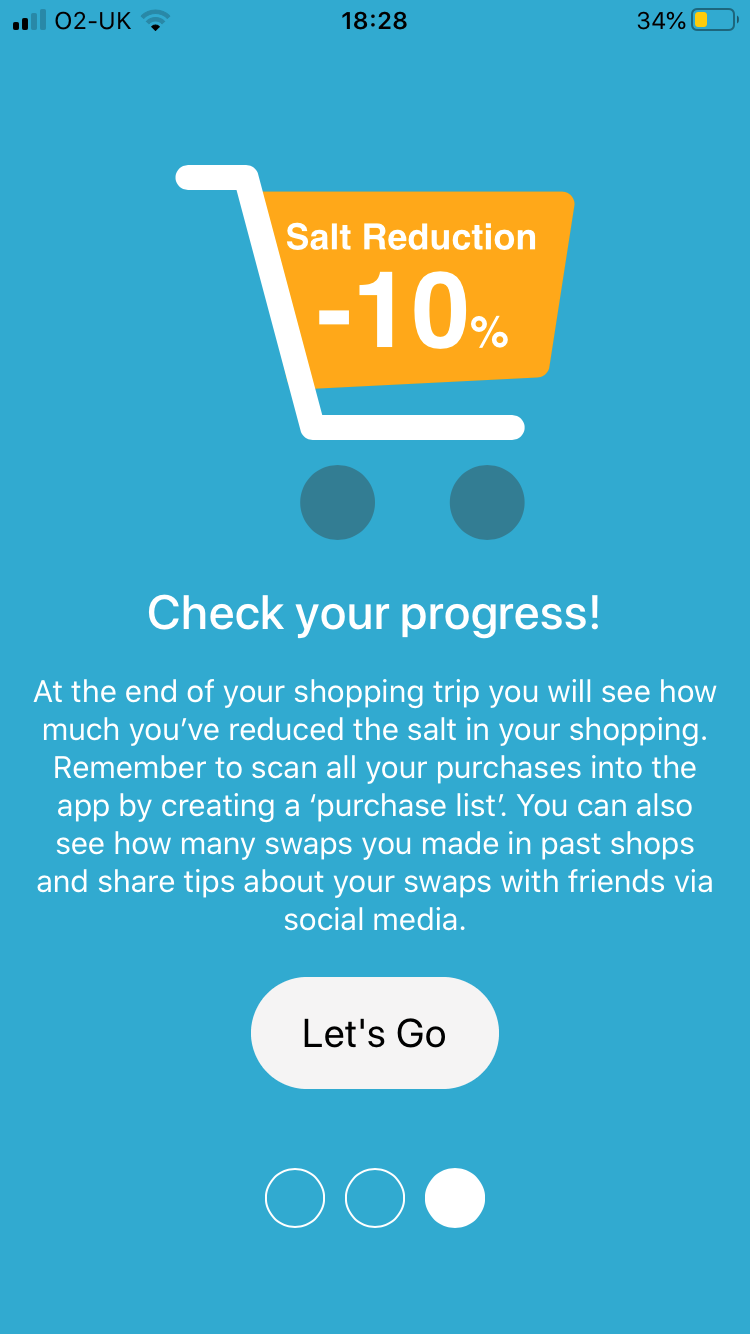


**Set a goal and use the app in store**

**
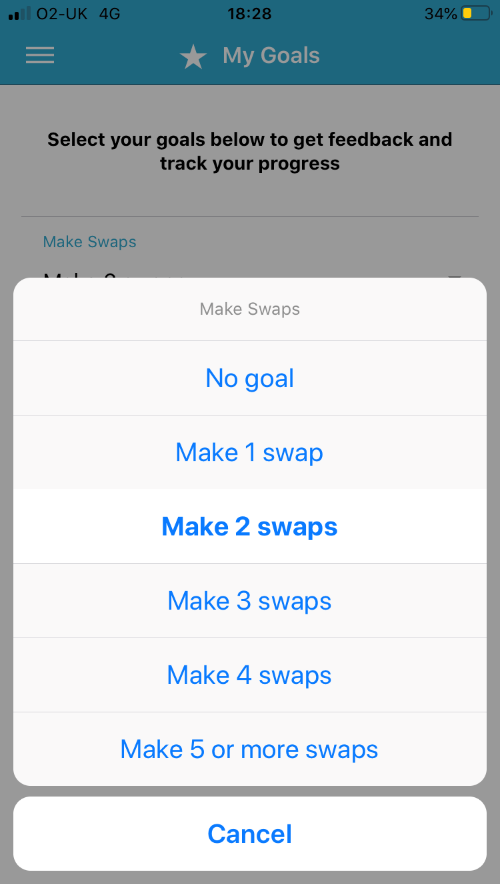

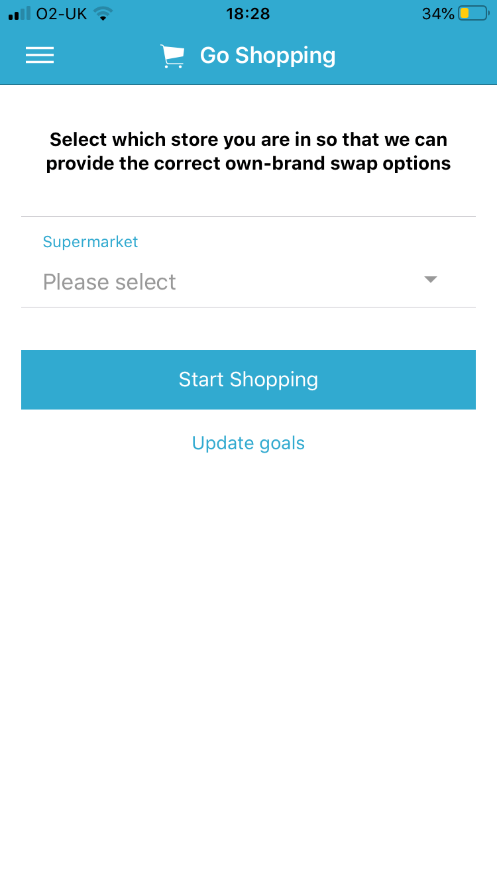
**


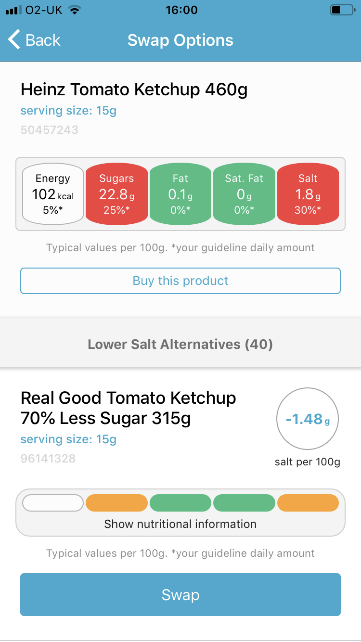
**
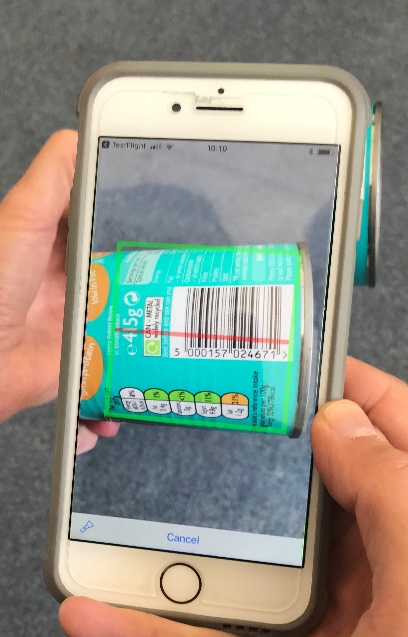
**


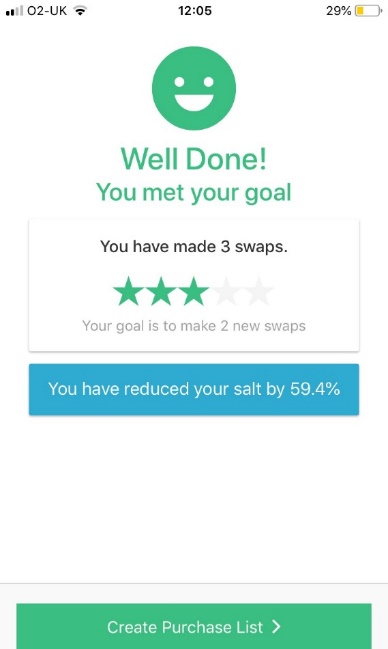

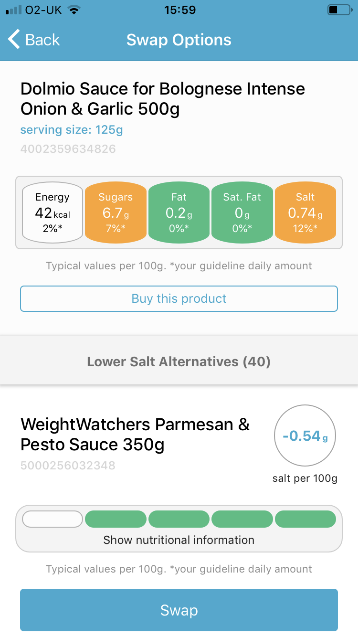


**SaltSwap app database**

The SaltSwap app is underpinned by a database of products available in UK grocery stores, with detailed nutrient data. This database was sourced from two key sources: a commercial organisation that collates product and nutrient data for several UK retail supermarkets (Ada, Sainsbury’s and Tesco) [41]; the app used a snapshot of store data taken in 2017; and The George Institute for Global Health [42] who have created a food and nutrient database of products (Foodswitch database), primarily crowd-sourced, from UK supermarkets including Tesco, Sainsbury’s, Morrison’s, Lidl, Aldi, Iceland, Waitrose, The Co-operative, and Marks & Spencer’s (extract taken from May 2018).

The SaltSwap database contained 95,290 unique products. All products in the app database are categorised according to a defined schema of product group (i.e. a wider selection of products) and category (i.e. from a narrower subset of products). Product categorisation utilised the product categories of the source databases and these were mapped to the new schema. An example product group and associated categories is group*: breakfast cereals;* categories*: hot cereals, ready-to-eat cereals, muesli & granola and breakfast cereal variety packs.* Suggested alternatives are drawn from the same group (the first ten results presented) followed by alternatives which match the same product group and category. Offering alternatives from the wider product group increases the variety of lower-salt products available and increases the potential magnitude of salt reduction through accepting a swap. The database of products was cleaned and entries with erroneous or incomplete data in essential fields were removed. The product data used in the SaltSwap app is not publicly available and, at the time of publication of this article, is likely to be out of date and is therefore not provided.
