# Supplementary material for: A Mobile Health Salt Reduction Intervention for People With Hypertension: Results of a Feasibility Randomized Controlled Trial
Source: JMIR Mhealth Uhealth. 2021 Oct 21;9(10):e26233. doi: 10.2196/26233 (PMC8569539; doi:10.2196/26233)

# Appendix 3: ShopScanner app.

The ShopScanner app, participants registered a user account, using their email, a chosen username and password.

**Instructions**

Scan the barcode of each product you buy using your phone camera.

Simply click on ‘Scan’ and hold the phone with the camera facing toward the product barcode and your camera will automatically recognise and scan the barcode. You may need to allow the camera to focus on the barcode for a few seconds.

After each item, click, ‘Scan’ to scan the next item.

Scan all items you purchase in your shopping trip. If they don’t have a product barcode, or the app doesn’t recognise the barcode, you can enter item manually by clicking the ‘Enter Manually’ button. When you have scanned or entered all products click ‘Finish Purchase List’ and your shopping (list of purchased items) is recorded. No personal data or cost data is collected.


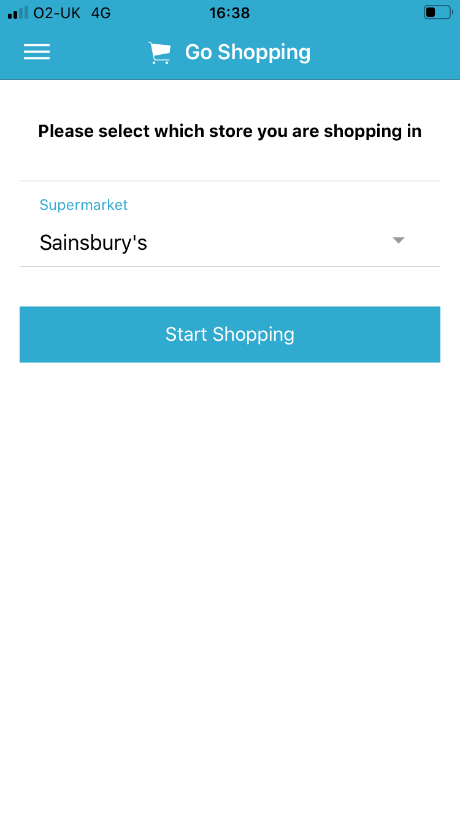

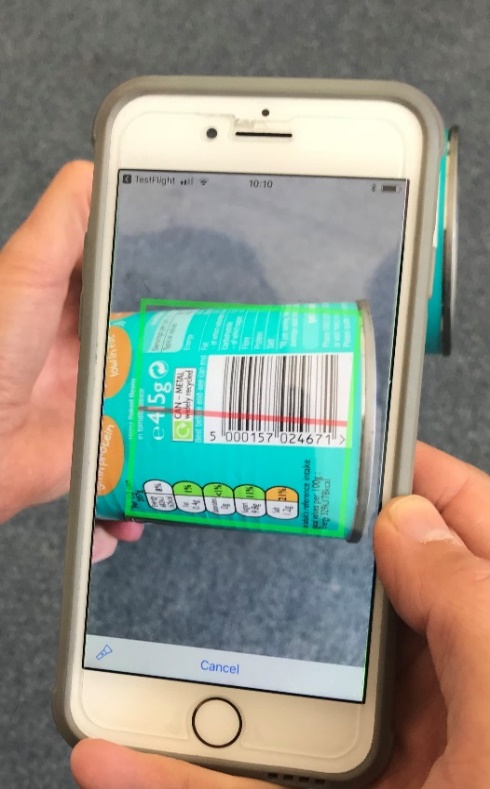


#
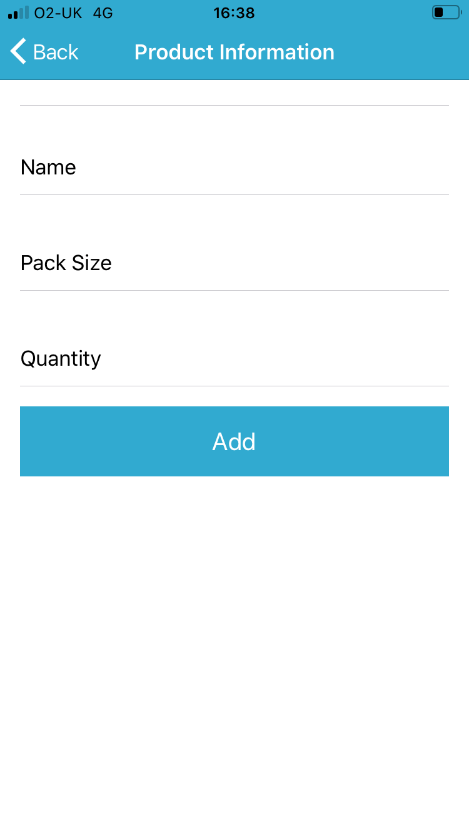

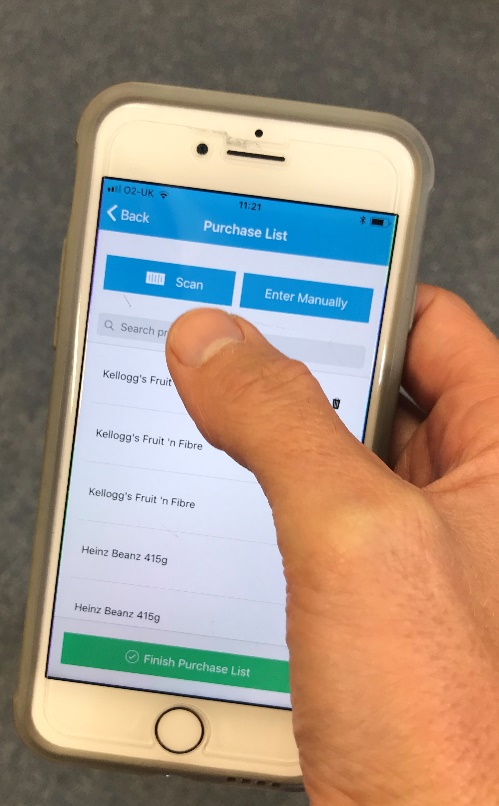

Supplement: Multimedia Appendix 3 [file mhealth_v9i10e26233_app3.docx]
