# Supplementary material for: A Mobile Health Salt Reduction Intervention for People With Hypertension: Results of a Feasibility Randomized Controlled Trial
Source: JMIR Mhealth Uhealth. 2021 Oct 21;9(10):e26233. doi: 10.2196/26233 (PMC8569539; doi:10.2196/26233)
Supplement: Multimedia Appendix 4 [file mhealth_v9i10e26233_app4.docx]

# Appendix 4. Qualitative methods and outcomes

Supplementary Table 5: Lincoln and Guba's Evaluative Criteria for ‘Trustworthiness’

| **Quality criteria** | **Description** | **Application** |
| --- | --- | --- |
| Credibility | Confidence in the 'truth' of the findings including triangulation, peer-debriefing, negative case analysis, member-checking, prolonged engagement, persistent observation and referential adequacy. | Not all these techniques were employed however, we used purposive sampling to include information rich cases, and a broad spectrum of cases across geographical areas, gender and age and timing within the intervention period. Direct observations were supplemented with one-to-one interviews to capture both naturally occurring and generated data on the topic. To avoid unconsciously steering the interview to generate data to support the SaltSwap intervention, we used a well-scripted topic guide with example questions, to ensure a broad range of topics were covered consistently across interviews and actively encouraged negative comments as well as positive. |
| Transferability | Showing that the findings have applicability in other contexts. Transferability can be achieved through, ‘thick description, described by Lincoln and Guba (1985) as a way of achieving a type of external validity.  By describing a phenomenon in sufficient detail one can begin to evaluate the extent to which the conclusions drawn are transferable to other times, settings, situations, and people. | We used codes that were close to the content of the data, in language and meaning where possible, and also used some interpretative codes to adequately capture phenomena. We sought out similarities and differences across accounts to ensure different perspectives were represented and included verbatim quotes of participant responses to support the constructs and findings reported in results. We aimed to present a fair representation of the data, not solely based on the frequency of reporting of similar views but with the aim of presenting the full breadth of views or experiences reported. |
| Dependability | Showing that the findings are consistent and could be repeated e.g. using ‘inquiry audit’ which involves having a researcher not involved in the research process examine both the process and product of the research study. | The lead author, who conducted the think aloud session and interviews discussed the coding framework with co-authors not directly involved in the data collection, before coding and after the first iteration. Example transcripts were reviewed and discussed, together with the developing coding framework, to determine how these could be grouped into themes and the evolving interpretation. |
| Confirmability | A degree of neutrality or the extent to which the findings of a study are shaped by the respondents and not researcher bias, motivation, or interest. Techniques to establish this include using an audit trail, triangulation of data and considering researcher reflexivity. | Codes were described in detail to ensure they were well defined and consistently applied. We monitored and recorded the decisions made during analysis to ensure consistent interpretation and enable reflection on the implications of these decisions on the reported outcomes. We recorded all iterations of the coding framework, as well as documenting reasons for amendments.  Qualitative data was triangulated with quantitative data where possible e.g. on use of the SaltSwap app.  The interviewer and lead author, considered how their own personal characteristics, position, relatedness to the research, and beliefs which were likely to have affected the data collection, analysis and interpretation, and took deliberate steps to mitigate this impact such as focussing on positive and critical aspects, taking care not to reinforce their own beliefs through ad-hoc comments to participants, and documenting the rationale for interpretation of data. |

Supplementary Table 6. Characteristics of think-aloud participants (n=12)

| **ID** | **Age (years)** | **Gender**  **(M/F)** | **Ethnicity^a^** | **Diagnosed**  **hypertension** | **Household**  **size** | **Shop**  **frequency^b^** | **Think-aloud**  **(week)** | **Total duration (min)** |
| --- | --- | --- | --- | --- | --- | --- | --- | --- |
| **0205** | 68 | F | WB | Yes | 2 | 1x | 3rd | 40 |
| **0206** | 64 | M | WB | Yes | 3 | >1x | 2nd | 70 |
| **0207** | 58 | M | WB | No | 3 | 1x | 2nd | 63 |
| **0218** | 76 | F | Ukn | Yes | 1 | >1x | 3rd | 37 |
| **0309^c^** | 71 | M | WB | Yes | 1 | 1x | 6th | 19 |
| **0313** | 57 | F | A | Yes | 2 | 1x | 8th | 48 |
| **0319** | 69 | F | WB | Yes | 3 | 1x | 7th | 84 |
| **0403** | 70 | M | WO | Yes | 2 | >1x | 6th | 48 |
| **0408** | 65 | M | WB | Yes | 1 | >1x | 6th | 38 |
| **0410** | 74 | F | WB | Yes | 2 | >1x | 5th | 60 |
| **0415** | 71 | F | WB | Yes | 2 | >1x | 6th | 34 |
| **0416** | 57 | F | WB | No | 2 | 1x | 5th | 40 |

^a^ White British (WB); Prefer not to say/unknown (Ukn); Other Asian (A); White other (WO)

^b^ Once a week; (1x) More than once a week (>1x)

^C^ Attended only the post-shopping interview due to participant time constraints on the day

Supplementary Table 7. Qualitative themes

| **Categories & themes** | **Description** | **Examples** |
| --- | --- | --- |
| **The intervention** | | |
| **Theme 1.**  **The intervention overall & impact on knowledge, motivation, intentions** | Describing their knowledge, current, previous of new knowledge from the study and their motivation to change their salt intake (intervention participants only). Descriptions or comments on their experience of the HCP advice component of the intervention.  Factors that motivate people to reduce salt intake or beliefs that negatively impact their motivation and willingness to change, and how they justify their choices. It includes emotions described when talking about shopping or diet, as emotion is a key influencer of motivation.  This also includes the role of feedback and experiences/comments about behavioural regulation techniques such as action planning, self-monitoring and goal setting.  . | *“I have been quite surprised on how high in salt breads are. That’s been a bit of an eye opener.” (ID0205, female, 68yrs)*  *“Well I hadn't realised that there were crisps that were lower in salt…But I've been really shocked at how much salt some food has in...and I mean the [store] ready meals.” (ID0410, female, 74yrs)*  “since been into this programme, has it made me look at different things in a different way, in a different manner? Yes, I suppose it has…I have decided now, like I said, is that to look at the way we do things – don’t put salt in my cooking now” (ID0206, male, 64yrs)  *“And strangely enough, you do get used to it. It's like not having sugar in your tea. Yes, I suppose it's a bit bad in some ways, but if it hasn’t affected the flavour and what I can actually do with something, then it's absolutely fine.” (ID0205, female, 68yrs)*  *“Yes, yeh, I think I could continue. What I want to do though is wait and find out what the blood pressure is... and I would be interested to see if it remains the same or whether it has changed....And if the... see the results of the urine test at the end of the study to see if the salt content has decreased there…it would be interesting because it does confirm that you’ve done the job well.” (ID0403, male, 70yrs)*  *“Well, I mean that was quite interesting because she was talking about targeting specific things each week.* “And she said, 'Well, if you kind of concentrate on a couple of things, and then you get into the pattern of doing those and knowing what to look for, then the following week you move on to something different.' And I think that was quite sensible advice really” *(ID0410, female, 74yrs)*  *“We sort of set a goal, which is why I've changed bread.” (ID0207, male, 58yrs)* |
| **Theme 2.**  **Using the SaltSwap app** | General comments and observations about the SaltSwap app. | *“I think my last shop, some of the sort of snacky crisp type things. They were like a ripple type crisp, and I was surprised how much salt was in that. And in fact, I don’t think I bought them in the end. Because I thought, 'Wow, I thought these were supposed to be, you know, healthy.'” (ID0206, male, 64yrs)*  *“Things such as the crackers, swapping them, corn crackers for lighter ones. That was a really interesting find.” (ID0319, female, 69yrs)*  *“because I have found that the bacon, the app was good for finding low salt bacon” (ID0410, female, 74yrs)*  *“I think it's a gadget which I'm comfortable using that’s made me more conscious of looking at the salt levels where normally, I would look at sugar levels more than salt” (ID0206, male, 64yrs)*  *“I did it at home for [store] and found out there was an alternative for [brand] pickles. With less salt. So, we got that this morning…” (ID0403, male, 70yrs)* |
| **Theme 3.**  **App barriers** | Barriers to using the Saltswap app or issues experienced when using it including functionality and usability. | *“where it gives you as an indication to use a different brand or a different make or a different product slightly, it's... the product's not been available in the store.” (ID0206, male, 64yrs)*  *“the recipe I used crumbles feta cheese on the top. Now, a low salt alternative came out as a sort of cream feta. Which just landed up as a revolting looking mess” (ID0205, female, 68yrs)*  ***“****I think the worst thing is the difficulty of trying to manage a mobile phone in one hand, your shopping list in another, pushing a trolley and negotiating round people. And then once you’ve got... it's using the app – the most frustrating thing is if it doesn’t come up, the barcode, isn't recognised.” (ID0205, female, 68yrs)*  *“I found was just requiring too much time and too much effort, where you come shopping and you don’t expect to be using your brain to analyse everything.” (ID0415, female, 71yrs)* |
| ***Salt consumption and purchase behaviours*** | | |
| **Theme 4.**  **Behavioural change** | Examples or discussion of changing their shopping or diet behaviour, including both intervention and control group participants. | *“It's making me definitely take something that’s got less salt, whereas I might not definitely take something with less fat. It just makes you... I can't remember what it is was I looked at now, but something had forty nine percent of your daily intake of salt. No way.” (ID0415, female, 71yrs)* |
| **Theme 5.**  **Barriers & facilitators** | Barriers to eating less salt or more healthily in general. | *“Yoghurts – I usually try and go for low sugar or fat free or both. I have found though that when I've been finding lower salt ones the levels of fat and sugar have been higher.” (ID0205, female, 68yrs)*  *“So, I think it is about how much time, making it easy and... but yeh, no, I think for us it's not been a big deal changing; it's just been interesting.” “So, I would rather do something beforehand, make my choices beforehand and then go in with my revised list” (ID0416, female, 57yrs)*  *“the problem was with the salt swap that the ones with less salt were not as tasty as those with more salt” (ID0403, male, 70yrs)* |
| ***The wider environment*** | | |
| **Theme 6.**  **The store environment** | How the grocery store environment shapes opportunities for healthy choices. Including comments on how the wider environment supports dietary change for salt reduction (or not) | *“I think there's too much choice. I absolutely love it when I go somewhere and I can go into a small supermarket and there's not the choice” (ID0205, female, 68yrs)*  *“Now we used to buy the deals here of different sorts of ham…but when I tried to do a salt swap on them it was quite difficult to find an alternative.” (ID0403, male, 70yrs)*  *“People are obviously very clever at making it look very appealing. And I think they're quite clever at selling something that isn't quite honest…[in terms of] the salt and the sugar” (ID0205, female, 68yrs)* |
| **Theme 7.**  **Nutrition labelling** | Description of use of nutrition labelling or barriers to use. Includes their experiences of labelling and their beliefs about and understanding of labelling as well as their knowledge about labelling and use of it. | *But there was other products on the shelf, and I basically looked at them and I actually looked at the label. Purely on the pretence of having a look of what the salt content is. And I've found that these other products, not only were they slightly cheaper but they had less salt. (ID0207, male, 58yrs)*  *“This is what I find quite difficult when some measurements are given in hundred grams and some are... So, but I mean to stand there and do all that I'd need a calculator as well to work out, you know” (ID0205, female, 68yrs)* |
| **Theme 8.**  **Responsibility** | Comments about personal, retailer or government responsibility for people making healthy choices. | *“let's stop being manipulated by these big supermarkets that basically encourage you to buy cheap or buy what you don’t really want because there's a special offer on. So, basically it's encouraging people to not eat healthy.” (ID0207, male, 58yrs)*  *“I think…for people to lower their salt content, it has to come from the government; it has to be through the manufacturers. To actually make a big difference…yeh. So, I think that would be the biggest benefit ” (ID0218, female, 76yrs)*  *“there's going to be a certain percentage of them who would be led by advertising and won't be proactive about a healthy diet. And so, I think there has to be legislation and the supermarkets to be responsible.” (ID0403, male, 70yrs)* |
| **Theme 9.**  **Support** | Comments about the availability and quality of support from healthcare providers or others to help people make healthy choices with their diet. | *“No, I don’t think, no. Not enough support, yeh.” (ID0313, female, 57yrs)*  *“I think from a point of view of the surgery, you can go in and they say, 'You’ve got high blood pressure, you'll have to take tablets for the rest of your life.' If they told you these are some suggestions to reduce your blood pressure, then I think that’s a good place for it to come from.” (ID0415, female, 71yrs)* |
