# Supplementary material for: A Mobile Health Salt Reduction Intervention for People With Hypertension: Results of a Feasibility Randomized Controlled Trial
Source: JMIR Mhealth Uhealth. 2021 Oct 21;9(10):e26233. doi: 10.2196/26233 (PMC8569539; doi:10.2196/26233)
Supplement: Multimedia Appendix 5 [file mhealth_v9i10e26233_app5.docx]

# Appendix 5. Process outcomes.

*Recruitment rate:* out of 2028 participants invited across five GP practices, 107 were assessed for eligibility, a response rate of 5%; and 50 were consented and enrolled (overall recruitment rate 2.5%). Three enrolled participants failed to provide all baseline data. Recruitment rate per month was not collected.

*Acceptability to healthcare professionals*: Healthcare professionals described the SaltSwap intervention as something that was within their role and skillset, comparing it to typical health promotion work. They felt confident and capable of delivering it, especially with the training provided and reported that the SaltSwap booklet helped them to deliver the advice sessions and provided them with new knowledge as well as their patients. They thought that the level was appropriate, participants engaged in the discussion and the behavioural components were useful. Some healthcare professionals noted that it might be challenging to implement this in routine primary care due to time limitations. The main barrier raised was experiencing technical issues downloading the app during the advice session (e.g. insufficient space to download the app, forgotten app store password, lack of data/internet to download it on site).

*Feasibility of data collection:* 24-hour urine samples were collected for all 47 randomised participants at baseline and for 43 participants (96%) of the 45 participants who attended the follow-up assessment. Participants recorded data in the study app for 97% of shopping trips that were recorded by receipt. In these shopping visits, 96% of purchased products (as documented on receipts) were recorded in the app. Across all participants, 42% of purchase products recorded in the app were entered manually, therefore were missing product nutrient data. Analysis of product categories for manually entered products showed that nearly half of these products were fresh fruit or vegetables, alcohol or non-grocery items and would be excluded from the analysis of the salt content of purchased foods.

Supplementary Table 3. Number of products purchased and recorded in the Shopscanner or SaltSwap apps, and missing data.

| **Purchased salt data** | **Control** | **SaltSwap** | **Total** |
| --- | --- | --- | --- |
| **Total number of products purchased per participant, baseline (m, SD)**  ***Missing data* (%)*** | 60 (29)  *40%* | 56 (35)  *37%* | 57 (32)  *38%* |
| **Total number of products purchased per participant, follow-up (m, SD)**  ***Missing data (%)*** | 45 (32)  *40%* | 29 (20)  *34%* | 34 (25)  *36%* |

*percentage of total products which lacked data on product salt content.

Supplementary Table 4. Process outcomes

| **N (%), unless otherwise specified** | **Total** | **Control** | **SaltSwap** |
| --- | --- | --- | --- |
| **Intervention acceptability** | **(n=47)** | **(n=16)** | **(n=31)** |
| Number of intervention participants who rate the nurse support as helpful or very helpful |  |  | 20 (71%), (n=28) |
| Number of intervention participants who rate the SaltSwap app as helpful or very helpful |  |  | 12 (43%) |
| Number of intervention participants who would use a free app like this |  |  | 19 (70%), (n=27) |
| **Use of the SaltSwap app** |  |  | **(n=29)** |
| Number of times the app is used throughout intervention period (i.e. number of shopping trips where it is used to scan products to identify swaps) (mean, (SD), % of all trips) |  |  | 8 (6), 75% |
| Number of products scanned (for swap), total (mean (SD)) |  |  | 39 (35) |
| Number of swaps accepted through the app, total (mean (SD)) |  |  | 3 (3) |
| Median salt reduction per swap accepted, g/100g (IQR) |  |  | -0.6 (-0.2 to -1.2) |
| The top food categories in which swaps were accepted |  |  | Bread, cheese, processed meats (accounting for 29% of all swaps recorded) |
| **Number of participants who set a goal in the app** |  |  | 27 (93) |
| **Knowledge and behaviour change** | **(n=44)** | **(n=16)** | **(n=28)** |
| Number of participants who increased their use of nutrition labels for salt | 35 (74) | 13 (81) | 22 (79) |
| Number of people who reported somewhat or strongly agree the study has improved their knowledge of salt & health | 39 (83) | 13 (81) | 26 (93) |
| **Dietary salt behaviours** |  |  |  |
| Swaps made to lower-salt foods |  | 8 (50) | 23 (82) |
| High-salt foods eaten less frequently |  | 11 (69) | 19 (68) |
| Reduced salt use in cooking |  | 9 (56) | 15 (54) |
| Reduced use of table salt |  | 5 (31) | 7 (25) |
| **Adherence to protocol** |  |  |  |
| Number of participants allocated to intervention arm, who attend the session |  |  | 31 (100) |
| Time between randomisation and intervention session (mean number of days) |  |  | 12 days |
| **Contamination** |  |  |  |
| Number of participants allocated to control, who access the SaltSwap app | - | 0 | - |
