# Supplementary material for: A Mobile Health Salt Reduction Intervention for People With Hypertension: Results of a Feasibility Randomized Controlled Trial
Source: JMIR Mhealth Uhealth. 2021 Oct 21;9(10):e26233. doi: 10.2196/26233 (PMC8569539; doi:10.2196/26233)
Supplement: Multimedia Appendix 6 [file mhealth_v9i10e26233_app6.pdf]

CONSORT-EHEALTH Checklist V1.6.2 Report	Manuscript Number	26233

(based on CONSORT-EHEALTH V1.6), available at [http://tinyurl.com/consort-ehealth-v1-6].

Date completed
10/1/2021 11:30:14
by
Sarah Payne Riches

A mobile health salt-reduction intervention for people with hypertension: results of a feasibility randomised controlled trial

TITLE

1a-i) Identify the mode of delivery in the title
Title: "A mobile health salt-reduction intervention for people with hypertension: results of a feasibility randomised controlled trial"
1a-ii) Non-web-based components or important co-interventions in title
Not in the title. Included in the abstract.
1a-iii) Primary condition or target group in the title
yes. "A mobile health salt-reduction intervention for people with hypertension: results of a feasibility randomised controlled trial"

ABSTRACT

1b-i) Key features/functionalities/components of the intervention and comparator in the METHODS section of the ABSTRACT
"randomised controlled trial of a mobile health intervention for salt reduction versus an advice leaflet (control). The intervention was developed using the Behaviour Change Wheel and comprised individualised, brief advice from a healthcare professional with use of the SaltSwap app"
1b-ii) Level of human involvement in the METHODS section of the ABSTRACT
The intervention was developed using the Behaviour Change Wheel and comprised individualised, brief advice from a healthcare professional with use of the SaltSwap app"
1b-iii) Open vs. closed, web-based (self-assessment) vs. face-to-face assessments in the METHODS section of the ABSTRACT
Abstract methods: Participants with an elevated blood pressure recorded in clinic, were recruited through primary care practices in the United Kingdom.
1b-iv) RESULTS section in abstract must contain use data
results for all primary outcomes. "Forty-seven participants were randomised. All progression criteria were met; follow up attendance (96%, n=45), intervention fidelity (81%, n=25) and app use (87%, n=27). "
1b-v) CONCLUSIONS/DISCUSSION in abstract for negative trials
"The intervention was acceptable and feasible to deliver within primary care; the trial procedures were practicable and there is sufficient signal of potential efficacy to change salt intake"

INTRODUCTION

2a-i) Problem and the type of system/solution
All points covered in introduction.
2a-ii) Scientific background, rationale: What is known about the (type of) system
yes. No room for further detail .

Does your paper address CONSORT subitem 2b?

"This paper describes the design of the SaltSwap intervention and reports the results of a randomised controlled trial incorporating qualitative and process outcomes. T

METHODS

3a) CONSORT: Description of trial design (such as parallel, factorial) including allocation ratio
"This feasibility study was an individually randomised, parallel, two-arm, controlled trial. "
3b) CONSORT: Important changes to methods after trial commencement (such as eligibility criteria), with reasons
no changes were made to methods
3b-i) Bug fixes, Downtimes, Content Changes
No significant changes were made after trial commencement
4a) CONSORT: Eligibility criteria for participants
"adults with a recent raised blood pressure reading (systolic blood pressure in the past two years above 130 mm Hg if currently prescribed a stable -dose anti-hypertensive medication, or above 140 mm Hg if not prescribed medication)
4a-i) Computer / Internet literacy
Desire to use an app to improve their diet was a criteria - not detailed in the manuscript but including explicitly in the protocol referenced
4a-ii) Open vs. closed, web-based vs. face-to-face assessments:
"Eligible participants were booked into a baseline study visit where a study researcher provided written study information and confirmed consent, in person";
4a-iii) Information giving during recruitment
"Eligible participants were booked into a baseline study visit where a study researcher provided written study information and confirmed consent, in person"
4b) CONSORT: Settings and locations where the data were collected
In 'procedures' section of methods
4b-i) Report if outcomes were (self-)assessed through online questionnaires
Throughout methods. Also more detail provided in referenced published protocol.
4b-ii) Report how institutional affiliations are displayed
Affiliations with university displayed on all trial documents including consent.
5) CONSORT: Describe the interventions for each group with sufficient details to allow replication, including how and when they were actually administered
5-i) Mention names, credential, affiliations of the developers, sponsors, and owners
Included in conflict of interest statement.
5-ii) Describe the history/development process
Included in manuscript and appendices
5-iii) Revisions and updating
Included in appendix detailing app development
5-iv) Quality assurance methods
No specific quality assurance was taken over and above pilot testing, which is discussed
5-v) Ensure replicability by publishing the source code, and/or providing screenshots/screen-capture video, and/or providing flowcharts of the algorithms used
Screen shots are provided and the function is detailed allowing replicability of the functionality, though the database is not replicable.
5-vi) Digital preservation
Screen shots are included along with detail so of the functionality
5-vii) Access
detailed in methods and in published protocol
5-viii) Mode of delivery, features/functionalities/components of the intervention and comparator, and the theoretical framework
Included in methods and appendices
5-ix) Describe use parameters
Instructions were delivered as part of the intervention face to face session. Detailed in methods.
5-x) Clarify the level of human involvement
throughout methods. examples removed as this document is too large to submit
5-xi) Report any prompts/reminders used
"SMS prompts were sent to remind participants to collect their shopping data if the app was not accessed within seven days and when the app was not used for ten days after first use."
5-xii) Describe any co-interventions (incl. training/support)
"The intervention comprised behavioural advice and support, provided by a healthcare professional (typically a nurse or healthcare assistant), in a 20 min face-to-face intervention visit. "
6a) CONSORT: Completely defined pre-specified primary and secondary outcome measures, including how and when they were assessed
throughout methods sections.
6a-i) Online questionnaires: describe if they were validated for online use and apply CHERRIES items to describe how the questionnaires were designed/deployed
not applicable as no online surveys
6a-ii) Describe whether and how “use” (including intensity of use/dosage) was defined/measured/monitored

"3)Use of the SaltSwap app: at least 50% of randomised participants use the SaltSwap app to scan products on at least one occasion in the first month"		
**6a-iii) Describe whether, how, and when qualitative feedback from participants was obtained**		
"Accompanied shopping and interview data were used in a qualitative analysis to explore the impact of the SaltSwap intervention on grocery shopping behaviour, use and acceptability of the SaltSwap app and experiences of attempting to reduce salt intake." "		
**6b) CONSORT: Any changes to trial outcomes after the trial commenced, with reasons**		
In 'procedures' section of methods		
**7a) CONSORT: How sample size was determined**		
**7a-i) Describe whether and how expected attrition was taken into account when calculating the sample size**		
" We calculated the sample size of 40 would be sufficient to estimate progression outcomes within acceptably narrow confidence intervals to enable robust testing of the trial methods"		
**7b) CONSORT: When applicable, explanation of any interim analyses and stopping guidelines**		
throughout methods sections.		
**8a) CONSORT: Method used to generate the random allocation sequence**		
"Participants were randomly allocated using a computer-generated allocation sequence, stratified by GP surgery, to allocate participants in a 2:1 ratio (intervention:control). "		
**8b) CONSORT: Type of randomisation; details of any restriction (such as blocking and block size)**		
"Participants were randomly allocated using a computer-generated allocation sequence, stratified by GP surgery, to allocate participants in a 2:1 ratio (intervention:control)"		
**9) CONSORT: Mechanism used to implement the random allocation sequence (such as sequentially numbered containers), describing any steps taken to conceal the sequence until interventions were assigned**		
"An independent researcher generated the random number sequence using an online random sequence generator and informed the principal investigator of intervention allocation for each participant. Participants and investigators were unaware of the treatment allocation prior to consent. "		
**10) CONSORT: Who generated the random allocation sequence, who enrolled participants, and who assigned participants to interventions**		
"An independent researcher generated the random number sequence" etc.. see above		
**11a) CONSORT: Blinding - If done, who was blinded after assignment to interventions (for example, participants, care providers, those assessing outcomes) and how**		
**11a-i) Specify who was blinded, and who wasn't**		
"Due to the nature of the intervention and trial procedures it was not possible to blind participants, clinicians delivering the intervention or the outcome assessor, to the treatment group."		
**11a-ii) Discuss e.g., whether participants knew which intervention was the "intervention of interest" and which one was the "comparator"**		
Participants were aware which was the 'intervention of interest'.		
**11b) CONSORT: If relevant, description of the similarity of interventions**		
Not relevant to this study		
**12a) CONSORT: Statistical methods used to compare groups for primary and secondary outcomes**		
Covered in "methods - Statistical analysis "		
**12a-i) Imputation techniques to deal with attrition / missing values**		
"Progression criteria analysis used data from all available randomised participants. Secondary outcomes were analysed on an intention-to-treat basis, using complete case analysis. "see methods for more details		
**12b) CONSORT: Methods for additional analyses, such as subgroup analyses and adjusted analyses**		
"We also examined the sensitivity of the results to confounding due to..."		
RESULTS		
**13a) CONSORT: For each group, the numbers of participants who were randomly assigned, received intended treatment, and were analysed for the primary outcome**		
Included in table 1. of results. The number of GP practices where patients were recruited is included in the methods.		
**13b) CONSORT: For each group, losses and exclusions after randomisation, together with reasons**		
Included in consort diagram		
**13b-i) Attrition diagram**		
Use of the app was one of the primary outcomes and use was included in process outcomes, so attrition not included separately		
**14a) CONSORT: Dates defining the periods of recruitment and follow-up**		
"Participants were recruited between October 2018 and April 2019." "Attendance at follow-up was 96% in total (94% in the intervention group and 100% in the control). Two participants did not attend follow up; one withdrew due to difficulty using the study app and one due to an unrelated health problem"		
**14a-i) Indicate if critical "secular events" fell into the study period**		
Not noted and not relevant within study period		
**14b) CONSORT: Why the trial ended or was stopped (early)**		
It was not stopped early		
**15) CONSORT: A table showing baseline demographic and clinical characteristics for each group**		
Table 1 shows this, in results		
**15-i) Report demographics associated with digital divide issues**		
Shown in table 1 of results and discussed in detail in discussion		
**16a) CONSORT: For each group, number of participants (denominator) included in each analysis and whether the analysis was by original assigned groups**		
**16-i) Report multiple "denominators" and provide definitions**		
Included in results section.		
**16-ii) Primary analysis should be intent-to-treat**		
"Secondary outcomes were analysed on an intention-to-treat basis, using complete case analysis"		
**17a) CONSORT: For each primary and secondary outcome, results for each group, and the estimated effect size and its precision (such as 95% confidence interval)**		
Included in results tables		
**17a-i) Presentation of process outcomes such as metrics of use and intensity of use**		
Included as process outcomes		
**17b) CONSORT: For binary outcomes, presentation of both absolute and relative effect sizes is recommended**		
included where applicable		
**18) CONSORT: Results of any other analyses performed, including subgroup analyses and adjusted analyses, distinguishing pre-specified from exploratory**		
included in results as per protocol and methods		
**18-i) Subgroup analysis of comparing only users**		
This is not included as not relevant		
**19) CONSORT: All important harms or unintended effects in each group**		
"One serious adverse event was reported during the study. The lead GP for the surgery concluded that this adverse event was unrelated to the study. The participant did not continue in the study or attend follow up, but no further action was taken."		
**19-i) Include privacy breaches, technical problems**		
There were none of these to report.		
**19-ii) Include qualitative feedback from participants or observations from staff/researchers**		
Qualitative outcomes were part of the core results		
DISCUSSION		
**20) CONSORT: Trial limitations, addressing sources of potential bias, imprecision, multiplicity of analyses**		
**20-i) Typical limitations in ehealth trials**		
Included in discussion section "Strengths and limitations"		
**21) CONSORT: Generalisability (external validity, applicability) of the trial findings**		
**21-i) Generalizability to other populations**		
Included in discussion e.g.' "Such respondents may differ from those we enrolled, meaning that the results here, indicating acceptability of the intervention and potential for changing salt intake behaviours, may not generalise to the wider population of people with hypertension"		
**21-ii) Discuss if there were elements in the RCT that would be different in a routine application setting**		
Discussed in discussion section e.g., "In clinical practice, brief interventions are likely to be given in consultation regardless of patients' inclination to reduce salt intake or interest in using an app."		
**22) CONSORT: Interpretation consistent with results, balancing benefits and harms, and considering other relevant evidence**		
**22-i) Restate study questions and summarize the answers suggested by the data, starting with primary outcomes and process outcomes (use)**		
Throughout discussion section		

**22-ii) Highlight unanswered new questions, suggest future research**		
Yes, in discussion section. e.g., "A full trial to assess the effectiveness of the SaltSwap intervention for reducing salt intake and blood pressure is warranted following improvements to the SaltSwap app highlighted as important during this feasibility study"		
Other information		
**23) CONSORT: Registration number and name of trial registry**		
"Trial Registration: ISRCTN 20910962 "		
**24) CONSORT: Where the full trial protocol can be accessed, if available**		
included as a reference to published protocol		
**25) CONSORT: Sources of funding and other support (such as supply of drugs), role of funders**		
Included all funding information		
**X26-i) Comment on ethics committee approval**		
"The study was reviewed and approved on the 17/03/2017 by the NHS Research Ethics Committee and the Health Research Authority (reference 17/SC/0098). "		
**x26-ii) Outline informed consent procedures**		
"Eligible participants were booked into a baseline study visit where a study researcher provided written study information and confirmed consent, in person"		
**X26-iii) Safety and security procedures**		
Adverse event reporting used: "All serious adverse events were reported during the trial. Participants were asked about adverse events at follow-up, or at the point of withdrawal from the trial. " Data governance and privacy policy were included in the informed consent process.		
**X27-i) State the relation of the study team towards the system being evaluated**		
all author conflicts and also stated conflict as owners of the app		
